# Orthogonally Functionalizable Redox-Responsive Polymer Brushes: Catch and Release Platform for Proteins and Cells

**DOI:** 10.1021/jacs.5c05856

**Published:** 2025-07-03

**Authors:** Aysun Degirmenci, Rana Sanyal, Harm-Anton Klok, Amitav Sanyal

**Affiliations:** † Department of Chemistry, 52949 Bogazici University, Bebek, Istanbul 34342, Türkiye; ‡ Center for Targeted Therapy Technologies (CT3), Bogazici University, Istanbul 34684, Türkiye; § 27218 Institut des Materiaux and Institut des Sciences et Ingenierie Chimiques, Laboratoire des Polymeres, Ecole Polytechnique Federale de Lausanne (EPFL), Batiment MXD, Station 12, Lausanne CH-1015, Switzerland

## Abstract

Polymer brushes engineered to “specifically capture” and “release on demand” analytes such as dyes, proteins, and cells find biomedical applications ranging from protein immobilization to cell death. Utilizing a disulfide-linker-containing monomer as a building block enables the fabrication of a redox-responsive polymer brush platform with the “catch and release” attribute. Herein, thiol-reactive redox-responsive polymer brushes are fabricated using a pyridyl disulfide-based monomer, and their postpolymerization functionalization is demonstrated via thiol–disulfide exchange reaction with thiol-containing dyes, (bio)­molecules, and cell adhesive ligands. After establishing reversible conjugation using a fluorescent dye and other model compounds, copolymer brushes postmodified with thiol-containing mannose demonstrated selective immobilization of concanavalin A in the presence of peanut agglutinin. In addition, a thiolated RGD peptide was conjugated to the side chain of polymer brushes to facilitate cell adhesion, followed by on-demand harvesting. To enable localized drug delivery to surface-adhered cells, orthogonal chain end and side chain functionalization using the thiol-Michael addition and thiol–disulfide exchange reaction, respectively, was used to conjugate the cell adhesive RGD peptide and the anticancer drug doxorubicin (DOX). On-demand DOX release and internalization by surface-bound cancer cells were demonstrated via cleavage of disulfide linkages in the presence of a reducing agent. This approach may provide an attractive methodology to deliver therapeutic agents precisely to specific cells.

## Graphical abstract

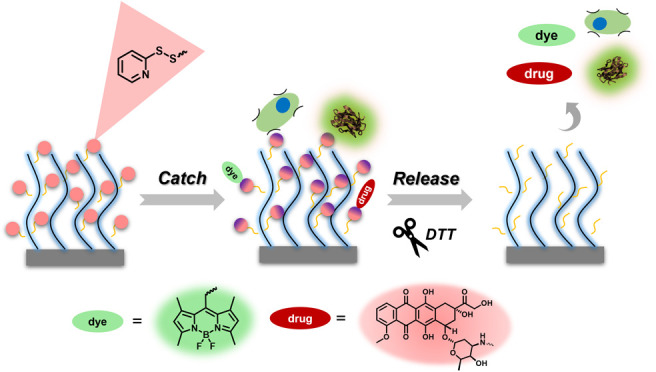

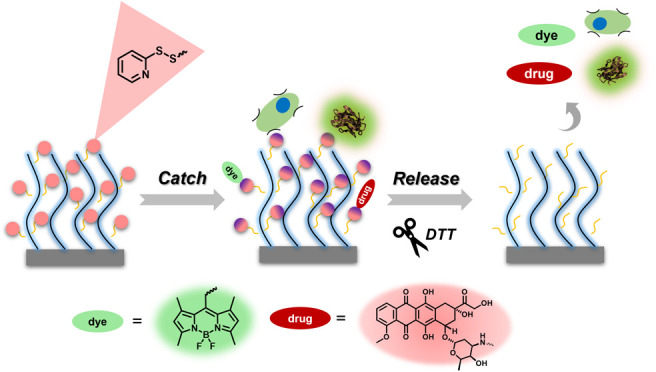

## Introduction

Thin polymer films incorporating reactive functional groups are attractive platforms to generate functional surfaces that play a critical role in the performance and properties of a wide range of (biomedical) materials.^1,2^ Among various methods for the preparation of the thin polymer films, the utilization of polymer brushes, which are surface-confined macromolecules where polymer chains are covalently tethered via one of the chain ends to a solid surface,^3^ has gradually increased in recent years due to their potential utilization for biomedical applications like biosensing, analyte detection, biomolecule immobilization, bioseparation, drug delivery, interfaces for cell attachment and growth, and antibacterial surfaces.^4–14^

Functional polymer brushes can be prepared in two ways: (i) direct surface-initiated reversible deactivation radical polymerization (SI-RDRP) using functional monomers and (ii) postpolymerization modification of brushes with reactive groups.^15^ Among these techniques, direct surface-initiated polymerization with functional monomers can often fabricate functional polymer brushes since a wide range of functional groups are suitable for this polymerization.^16^ However, a variety of functional groups are not compatible with direct surface-initiated polymerization as they can interfere with the propagating radical species during polymerization.^17,18^ Consequently, postpolymerization techniques have become of great value as a complementary approach to fabricating polymer brushes incorporating side-chain functional groups. Examples of side chain functional groups that have been incorporated into polymer brushes via postpolymerization modification reactions include azide,^19^ alkene,^20^ alkyne,^21^ NHS-activated ester,^6,22,23^ epoxide,^24,25^ thiolactone,^26^ furan,^27^ maleimide,^28^ aldehyde,^29,30^ and amine^31^ moieties.

Immobilizing biomolecules to solid surfaces mediated by the thiol functional group is one of the most widely used approaches in bioconjugation. For example, surfaces containing the maleimide functional group are widely used to immobilize thiol-bearing small molecules and biomolecules.^28^ As an alternative thiol-reactive group, the utilization of the pyridyl disulfide (PDS) group has gradually increased in recent years to provide the conjugation of thiolated molecules.^32^ In contrast to thiol-maleimide conjugation, the disulfide-thiol exchange reaction is reversible; therefore, this exchange reaction has been exploited from reversible conjugation of biomolecules to the fabrication of redox-responsive materials.^33^ To date, many examples of redox-responsive polymeric materials such as, copolymers,^34^ polymer aggregates,^35^ nanogels,^36–38^ micelles,^39–48^ and hydrogels^49,50^ have been reported. On the other hand, the fabrication of redox-responsive polymer coatings has been much less explored.^51–53^ Examples of redox-responsive micropatterned hydrogel coatings,^51^ polymeric thin films,^52,53^ and polymer brushes^54–56^ have been reported by thiol–disulfide exchange reaction. Still, most efforts involve the addition of the disulfide groups onto polymer brushes as a postpolymerization functionalization.

Herein, we demonstrate the fabrication of pendant pyridyl disulfide (PDS) bearing reactive redox-responsive polymer brushes via direct surface-initiated polymerization. The introduction of the PDS group allows reversible functionalization with thiol-containing organic molecules, fluorescent dyes, cell adhesive ligands, and chemotherapy drugs via a thiol–disulfide exchange reaction (Scheme 1). Moreover, reducing the thioester-based chain end groups paves the way for the secondary functionalization of polymer brushes. The surfaces fabricated via thiol–disulfide exchange reaction and orthogonal *click* reactions, such as the Michael addition reaction, are amenable to facile functionalization to tailor them for specific applications. As a proof of concept, we demonstrate that the polymeric interfaces designed here could be utilized to deliver drugs to cells in a localized manner by using a combination of cell-adhesive peptides and reversible conjugated drug. Although here we use a specific drug, the approach could be utilized to precisely deliver any molecule of interest to spatially localized cells.

**Scheme 1:**
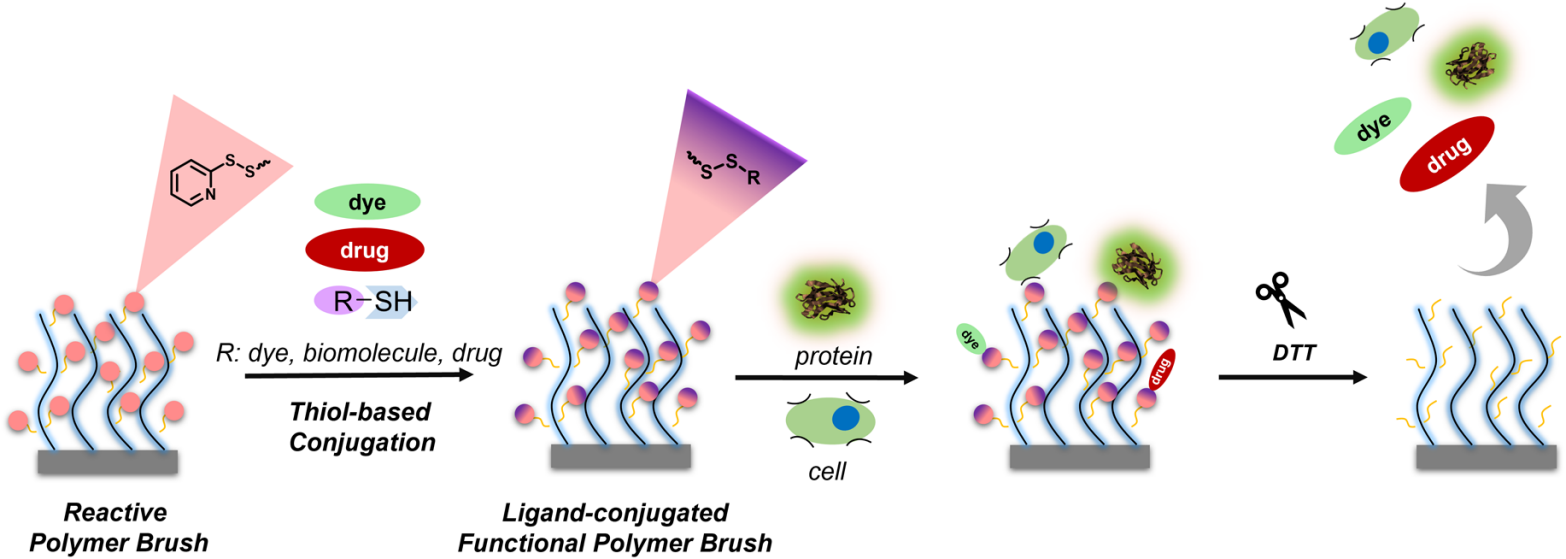
Fabrication and Functionalization of Pyridyl Disulfide-Based Thiol-Reactive Redox-Responsive Polymer Brushes

## Results and Discussion

### Preparation and Characterization of Pyridyl Disulfide-Containing Copolymer Brushes

Polymer brushes containing thiol-reactive pyridyl disulfide (PDS) side chain functional groups were obtained by using surface-initiated reversible addition–fragmentation chain transfer (SI-RAFT) polymerization (Figure 1a). Polymer brushes were prepared from phenyl thioester-based RAFT reagent-coated silicon surfaces using pyridyl disulfide ethyl methacrylate (PDSMA) and di­(ethylene glycol) methyl ether methacrylate (DEGMA) monomer in the presence of AIBN at 75 °C in DMF for 5 h. RAFT chain transfer agent (CTA)-coated silicon surfaces were prepared using the literature procedure.^17^ Briefly, cleaned Si/SiO_2_ surfaces were immersed in a trialkyl ether group-containing RAFT CTA solution. After immobilizing the RAFT CTA agent, surfaces were exposed to high-intensity UV light irradiation for 10 min using a photomask to obtain patterned copolymer brushes. Copolymer brushes generated from four different molar ratios of DEGMA/PDSMA in the feed (P2:90/10, P3:80/20, and P4:70:30) were synthesized and used in functionalization studies with thiol-containing molecules. In addition to these copolymer brushes, the corresponding P­(DEGMA) (P1) and P­(PDSMA) (P5) homopolymer brushes were also prepared. Polymer brush film thicknesses were determined by evaluating the step heights of cross-sectional profiles of micropatterned polymer brush samples, obtained using surfaces coated with patterned RAFT agent. By modulating the polymerization time, the film thickness of the polymer brushes could be controlled, as demonstrated in Figure 1c for the SI-RAFT copolymerization of a monomer feed containing 10 mol % PDSMA.

**Figure 1:**
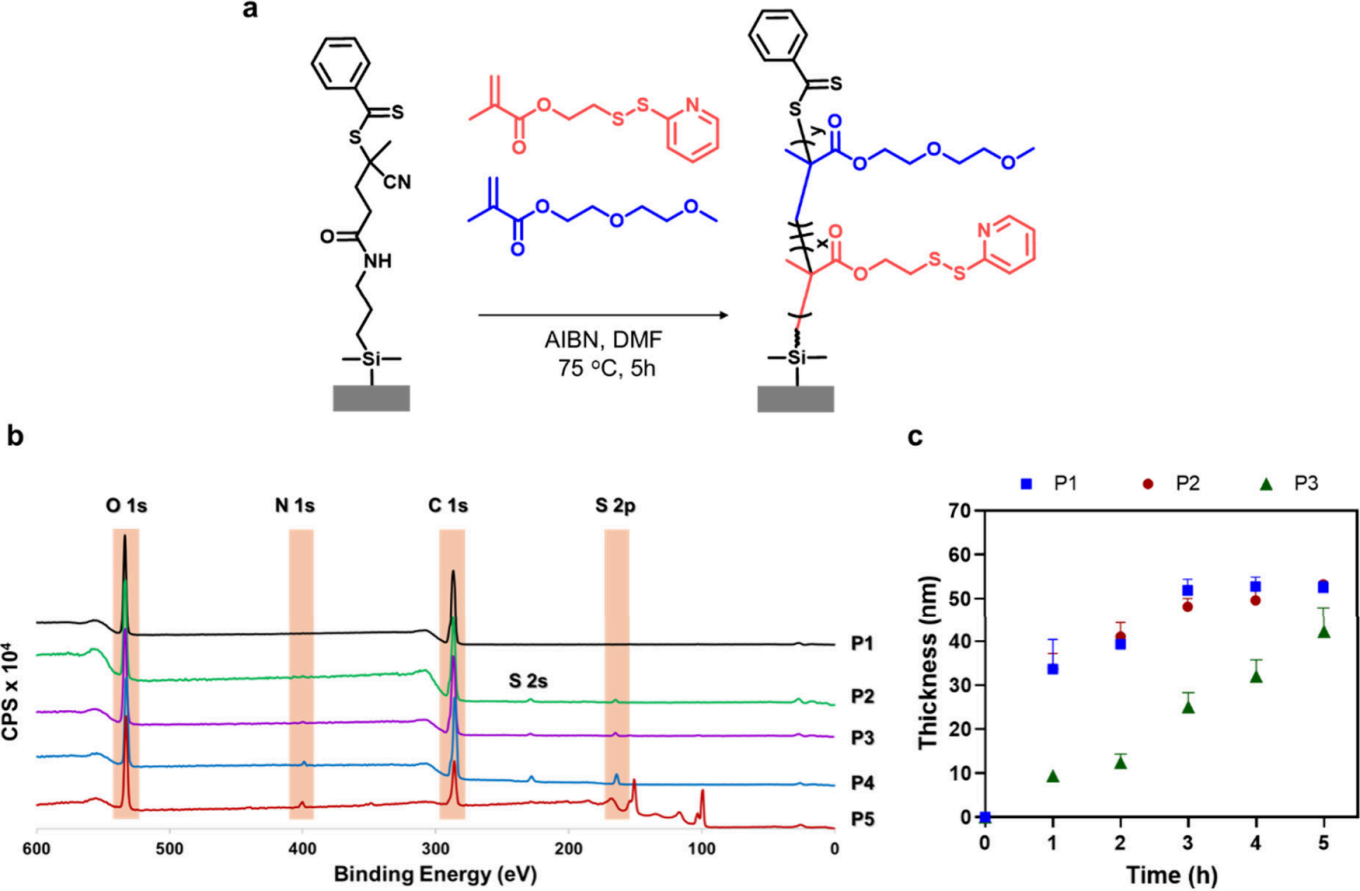
(a) Synthesis of PDSMA-functionalized copolymer brushes via SI-RAFT polymerization. (b) XPS survey spectra of polymer brushes P1–P5. (c) Evolution of brush thickness as a function of polymerization time for the synthesis of brushes P1, P2, and P3.

The P­[DEGMAy-co-PDSMAx] copolymer brushes were characterized by AFM, XPS, and water contact angle analysis. Table 1 summarizes the results of these experiments. AFM analysis of patterned brushes was used to determine the film thicknesses. Upon increasing the PDSMA content in the monomer feed, the film thickness of the polymer brushes obtained upon polymerization for 5 h decreases from ∼50 nm (P1 and P2) to ∼40 nm (P3) and ∼5–10 nm (P4 and P5) (SI Figure S1). In P1 (100% DEGMA) and P2 (10% PDSMA), fast initiation and rapid growth of polymer chains was observed. It was observed that as the PDSMA content increased, a notable decrease in polymer growth occurred. Nonetheless, as demonstrated later, the polymer brush incorporating 10% PDSMA was sufficient for the targeted applications. While the film thicknesses reported in Figure 1c and Table 1 are typical for surface RAFT polymerizations,^17,57^ the use of other surface-initiated controlled radical polymerization techniques may afford even thicker brushes. The composition of the P­[DEGMAy-co-PDSMAx] copolymer brushes was analyzed by using XPS analysis (Table 1 and SI Table S1). Figure 1b shows the survey XPS spectra of a P­(DEGMA) (P1) homopolymer brush, P­[DEGMAy-*co*-PDSMAx] copolymer brushes (P2–P4), and P­(PDSMA) homopolymer brush (P5). The nitrogen N 1s signal at 399.7 eV and the sulfur S 2p signal at 164.0 eV are evident for successfully incorporating the PDSDMA comonomer in polymer brushes. As shown in Figure 1b, the intensity of the N 1s and S 2p signals increases with an increasing mole fraction of PDSMA in the comonomer feed. In contrast, as expected, no N and S peaks are present in the survey scan of the P­(DEGMA) homopolymer brush. The composition of the copolymer brushes was determined using the XPS O 1s and N 1s signals and is summarized in Table 1. The results show that the composition of the copolymer brushes resembles that of the monomer feed. In addition to XPS, the polymer brushes were also characterized with water contact angle analysis (Table 1). Incorporating and increasing the PDSMA content in the polymer brushes is accompanied by an increase in the water contact angle compared to the more hydrophilic P­(DEGMA) homopolymer brush.

**Table 1:** Properties of Polymer Brushes (P1–P5) and Calculated Monomer Composition from XPS Based on the N/O ratio

Sample^a^	DEGMA/PDS (y/x) feed molar ratio	DEGMA/PDS (y/x) calcd. from XPS (N/O)^b^	Thickness (nm)^c^	Water Contact Angle^d^
**P1**	100/0	100/0	52 ± 2.0	63 ± 4
**P2**	90/10	93.3/6.7	53.7 ± 2.3	71 ± 3
**P3**	80/20	85.6/14.4	42.2 ± 5.0	72 ± 3
**P4**	70/30	80.95/19.05	10.3 ± 1.2	76 ± 3
**P5**	0/100	0/100	5.0	82 ± 4

a Polymerization was performed at 75 °C for 5 h.

b Determined by XPS analysis of non-patterned polymer brush surfaces.

c Determined by AFM analysis using micropatterned surfaces.

d Determined using nonpatterned surfaces.

The N 1s high-resolution spectra of polymer brushes P1–P5 are presented in Figure 2. The N 1s peak is not visible in the spectrum of the P­(DEGMA) homopolymer brush P1, suggesting that the polymer chains homogeneously cover the surface, thus masking the N atoms in the RAFT CTA. The spectra of copolymer brushes P2–P4 and that of PDSMA homopolymer brush P5 showed the expected N 1s peak at 399.7 eV belonging to the PDS group. As expected, the intensity of the N 1s peak increases with the increasing ratio of PDSMA monomer in the polymer brush. C 1s high-resolution spectra of polymer brushes P1 – P5 are given in SI Figure S2. The C 1s high-resolution spectrum of the P­(DEGMA) homopolymer brush P1 can be deconvoluted to three Gaussians at 288.9, 286.5, and 285.0 eV, corresponding to the C=O, C–O–C and C–C signals, respectively. Likewise, the C 1s high-resolution spectrum of the P2 brush can be deconvoluted to three Gaussians at 288.9, 286.4, and 285 eV, corresponding to the C=O, C–O–C/C–N/C–S, and C–C signals respectively. SI Figure S3 presents the S 2p high-resolution XPS spectra of polymer brushes P1–P5. While the P­(DEGMA) homopolymer brush P1 does not show any S 2p peak, these are visible at 164.2 eV in the spectra of brushes P2–P5, and could be deconvoluted into two Gaussians with the expected relative areas for two sulfur atoms at 165.1 eV (S 2p_1/2_) and 163.8 eV (S 2p_3/2_), S–S, C–S, respectively and data were similar to literature.^58^ As it was not possible to obtain PDS homopolymer brushes with thicknesses of more than 5 nm, the deconvoluted S 2p peak for brush P5 also features the signal from the dithioester group of the surface-bound RAFT agent.^17^ Similar to the N 1s signals, the S 2p peak intensity increased with the increasing ratio of the PDSMA monomer in the copolymer brush.

**Figure 2:**
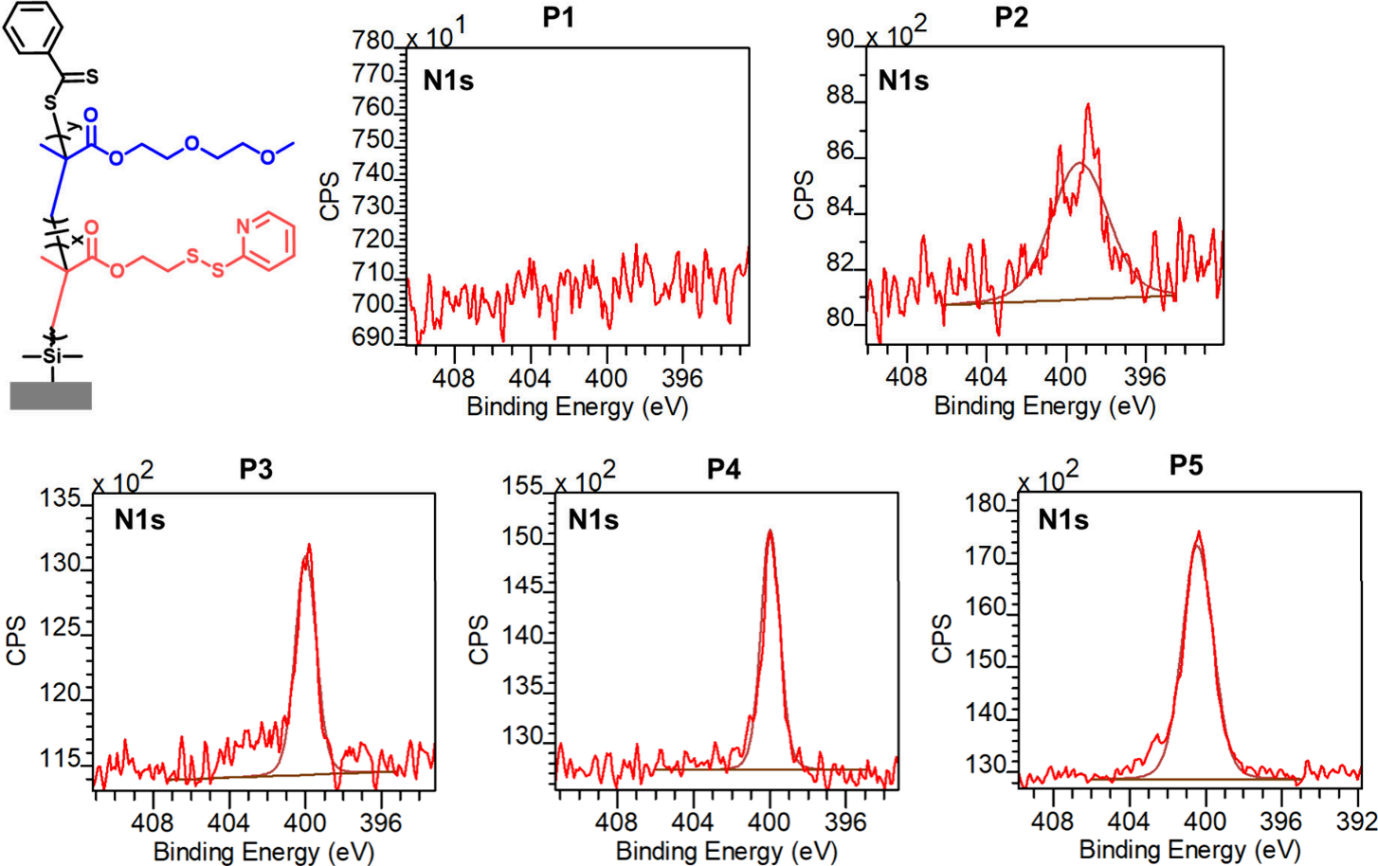
High-resolution N 1s XPS spectra of polymer brushes P1–P5.

After fabrication and characterization of PDS-containing polymer brushes, the attachment of a dye, biomolecules, or cells was investigated. The structural dispersity of polymer brushes fabricated using synthetic polymers is a significant property that affects their interfacial properties. Benetti and colleagues reported that side-chain heterogeneity (dispersity of side chains) with graft polymer leads to more hydrated, less adhesive, more lubricious and biopassive polymer films compared to homogeneous polymer films.^59^ Hoogenboom, Benetti and co-workers highlighted effect of side-group composition within polymer brushes on significant properties such as the hydration, main-chain flexibility and bioinertness.^60^ So, the investigation of accessibility of the PDS groups in these polymer brushes was undertaken to access the functionalizability of these interfaces.

### Functionalization of PDS-Containing Polymer Brushes

The PDS side chain functional groups of the copolymer brushes are amenable to modification by using the thiol–disulfide exchange reaction. To investigate this functionalization, a thiol–disulfide exchange reaction using a thiol-functionalized BODIPY-derivative was investigated (Figure 3a). A substrate modified with micropatterned copolymer brush P2 was immersed in a dye-containing solution (1 mg/mL) in the presence of a catalytic amount of acetic acid. As a control experiment, the P­(DEGMA) P1 homopolymer brush devoid of the thiol-reaction side chain was used and treated with the thiol-containing dye under the same conditions. After the functionalization, surfaces were rinsed with DMF and THF to remove unbound BODIPY and characterized using fluorescence microscopy. Green fluorescence suggested successful BODIPY dye conjugation onto the PDS-containing polymer brush (Figure 3b, 0 h), while the control surface did not reveal any fluorescence (Figure 3b, 0 h, inset). We were concerned about the possibility of the reaction of BODIPY-SH with the polymer chain end, so we treated the copolymer brush P1 with the thiol-containing dye. No attachment of the dye or the creation of a thiol group (as verified using treatment with TAMRA-maleimide) was observed. Thus, the dye attachment takes place through the thiol–disulfide exchange and does not result in any other side reactions (SI Figure S4).

**Figure 3:**
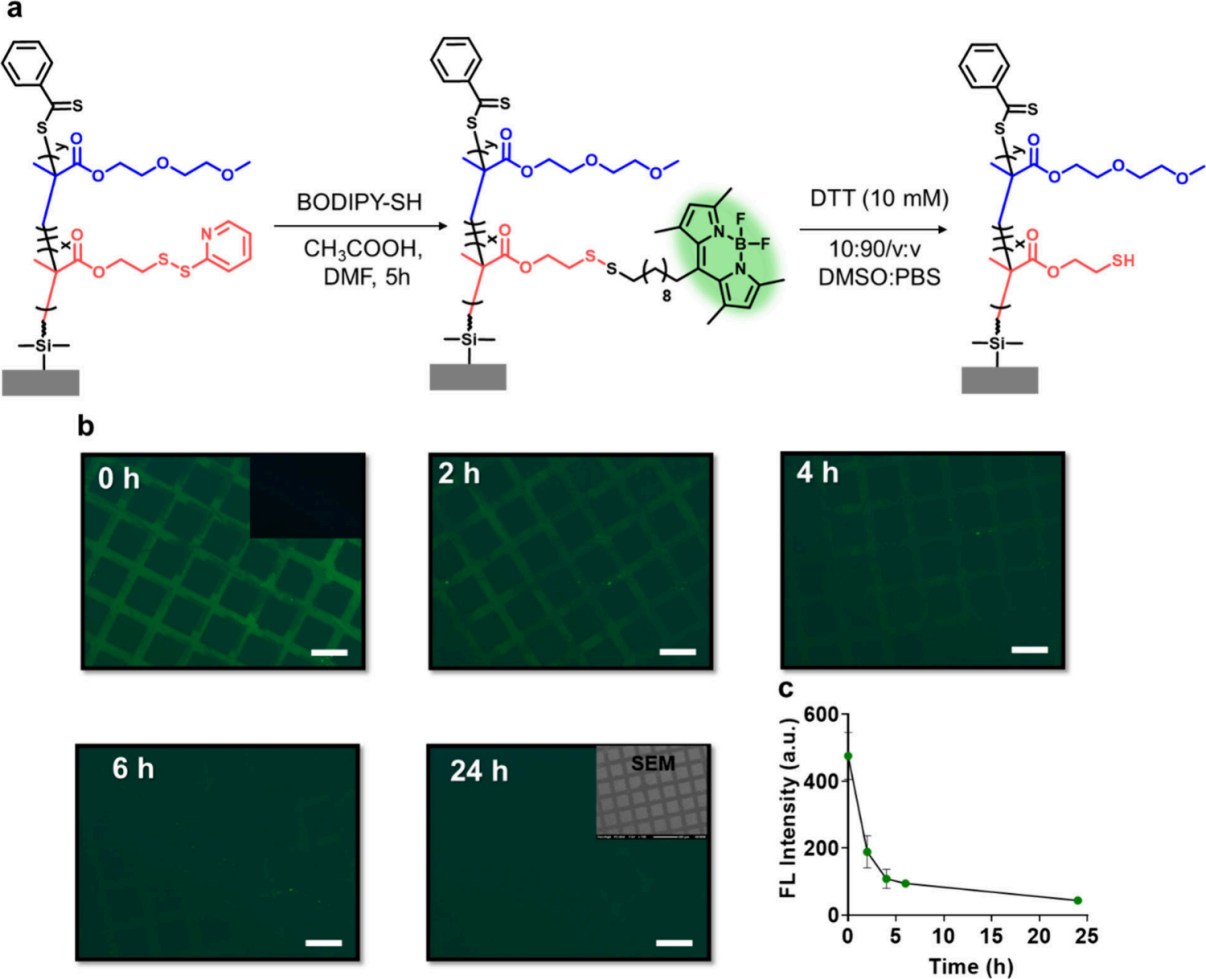
(a) BODIPY-SH immobilization on patterned P2 polymer brushes and cleavage of BODIPY with DTT from P2 polymer brush. (b) Fluorescence images of the surface during BODIPY release. The fluorescence microscope image of control surface is shown as an inset in the 0 h image. SEM image of the polymer brush was shown as an inset. (c) Time-dependent BODIPY release graph. The scale bar is 100 μm.

To establish the reversible nature of this conjugation, the BODIPY-conjugated copolymer brush P2 was treated with a 10 mM solution of dithiothreitol (DTT) in PBS (10% DMSO) to investigate the release of dye from the surface. At predetermined time points, the surface was removed from the DTT solution. A fluorescence image was recorded after washing, and the surface was reimmersed into the DTT solution (Figure 3b). The time-dependent release of BODIPY from the surface was observed (Figure 3c). As expected, the green fluorescence decreased due to the release of the dye through cleavage of the disulfide bonds in the presence of DTT. The cleavage efficiency of BODIPY dye from the polymer surface was determined as 91% by comparing fluorescence intensities of polymer brushes before and after DTT treatment.

After the reversible attachment of BODIPY-thiol to the PDS-containing copolymer brushes was demonstrated, another functionalization was investigated using trifluoromethyl benzyl mercaptan (TFBM). This particular mercaptan was chosen due to the presence of the trifluoromethyl group, which provides distinct peaks in FTIR and XPS analyses and enables following the functionalization process at the molecular level. The P2 brush surface was incubated with a solution of TFBM in the presence of the catalytic amount of acetic acid (Figure 4a). The postmodified polymer brush was characterized with FTIR spectroscopy, water contact angle analysis, and XPS. After modification with TFBM, symmetric −CF_3_ and asymmetric −CF_3_ stretching peaks at 1327 and 1067 cm^–1^, respectively, were observed in the FT-IR spectrum (Figure 4b).^61^ The successful postpolymerization modification was also supported with XPS analysis (Figure 4c,d). The existence of the F 1s peak at 688 eV showed modification of PDS groups with TFBM. Comparison of the N 1s and F 1s intensities in the high-resolution XPS spectra suggests that 95% of the PDS side chain functional groups have undergone the thiol–disulfide exchange reaction.

**Figure 4:**
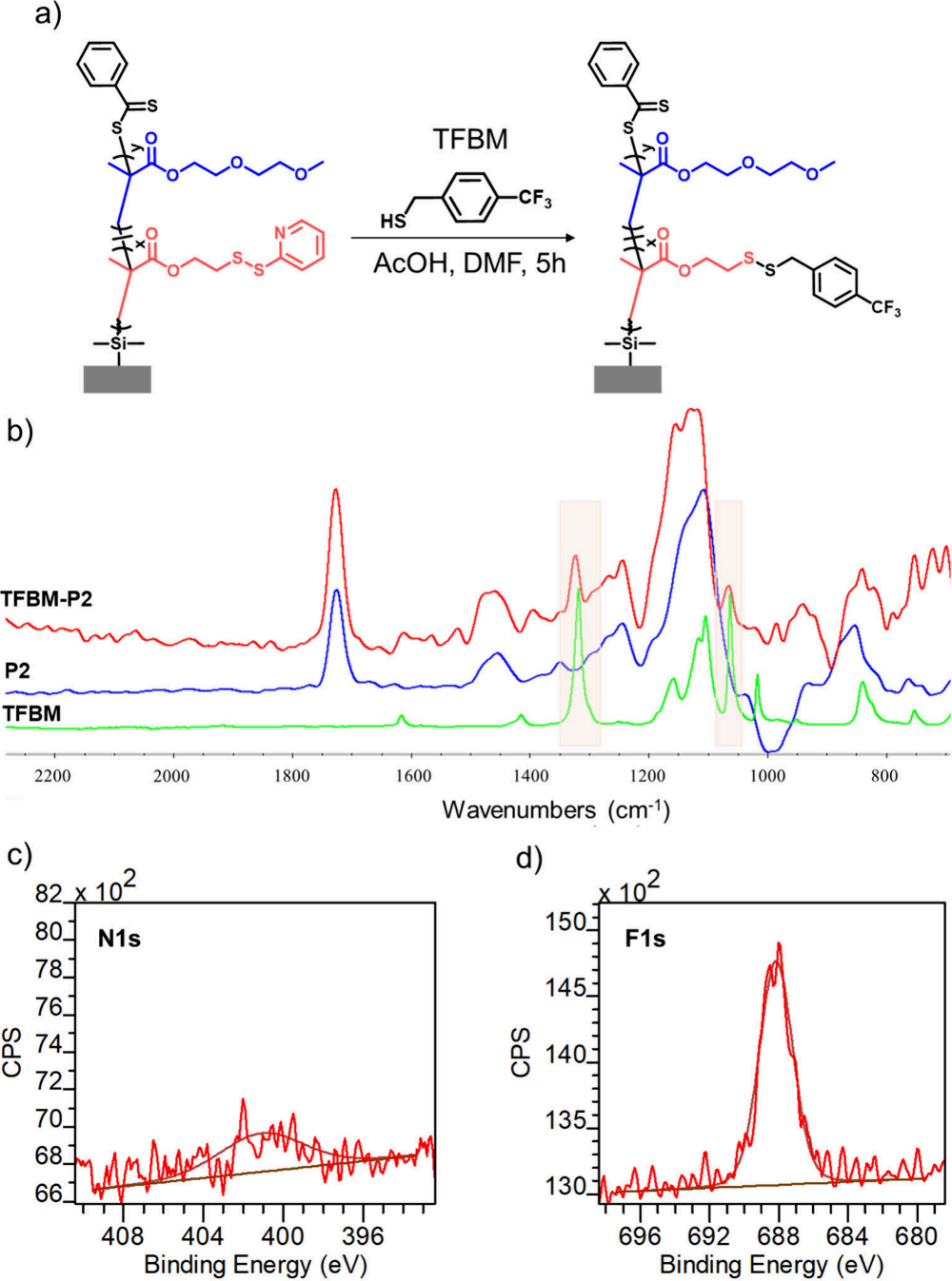
(a) Functionalization of the P2 brush with TFBM. (b) FTIR spectra of TFBM, P2 brush, and TFBM conjugated P2 brush. (c) High-resolution N 1s XPS spectrum of TFBM conjugated P2 brush. (d) High-resolution F 1s XPS spectrum of TFBM conjugated P2 brush.

### Orthogonal Functionalization of Polymer Brushes

Since the polymer brushes were prepared using SI-RAFT with the R group approach, thiocarbonate functional units were located at the top of the polymer chains on the surface. Postpolymerization modification methods would diversify the phenyl dithioester groups at the top of the polymer chains. In particular, phenyl dithioester functional groups can be reduced to thiol groups in the presence of an amine-containing reagent,^62^ which would pave the way for orthogonal modification of polymer brushes via thiol-maleimide chemistry. To test this, copolymer brush P2 (thickness, ca. 50 nm) was treated with 3-amino-1-propanol to generate thiol groups from phenyl dithioester units at the chain ends. Subsequently, the surface was treated with maleimide-bearing rhodamine dye (TAMRA-Mal, 1 mg/mL), and the success of the conjugation of rhodamine dye was characterized by fluorescence microscopy analysis. The efficiency of conjugation between TAMRA-Mal and the thiol-containing P2 brush was determined using Ellman’s assay. The thiol-containing P2 brush was treated with Ellman’s reagent (20 μL from 2 mg/mL) for 1h before and after the conjugation reaction with TAMRA-Mal. Ellman’s assay suggested that 97% of thiol groups on the P2 brush reacted with TAMRA-Mal dye. After that, the rhodamine-conjugated surface was immersed in a 1 mg/mL solution of BODIPY-SH in DMF in the presence of acetic acid to functionalize PDS groups on the side chains of the polymer brush using the thiol–disulfide exchange reaction (Figure 5a). After modification, dual-dye-conjugated polymer brush P2 was analyzed with fluorescence microscopy. Red fluorescence due to rhodamine and green fluorescence due to BODIPY confirmed that both dyes were successfully conjugated (Figure 5b). As a control experiment, a thiol-containing P2 brush was treated with a maleimide-devoid rhodamine B dye (1 mg/mL in THF) for 3 h, and then the surface was analyzed using fluorescence microscopy. Absence of any red fluorescence suggested that the rhodamine B dye does not undergo physical adsorption on the P2 brush (SI Figure S5). After orthogonal functionalization of the P2 brush with BODIPY and rhodamine dye, this surface was immersed in 10 mM DTT solution in DMSO to investigate BODIPY release. After 24 h of incubation at room temperature, the surface was analyzed under a fluorescence microscope. The disappearance of green fluorescence showed that BODIPY dye was cleaved from the surface in the presence of DTT (SI Figure S6).

**Figure 5:**
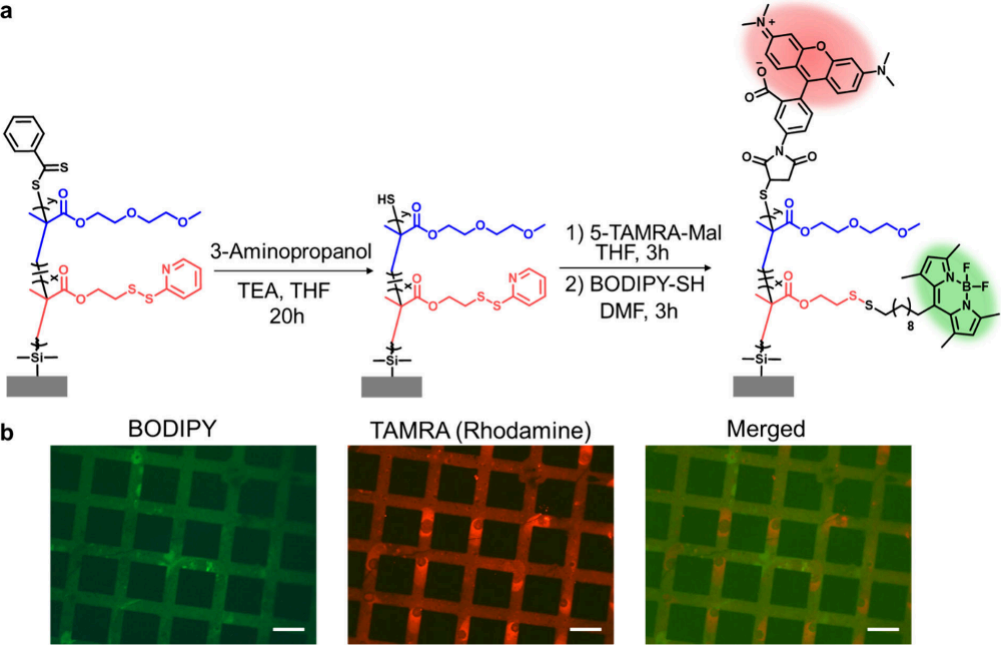
(a) Treatment with 3-amino-1-propanol to reduce dithiocarbonyl groups to thiols and immobilization of maleimide-containing rhodamine and subsequent BODIPY-SH conjugation. (b) Fluorescence images of rhodamine and BODIPY-conjugated P2 polymer brush. The scale bar is 100 μm.

### Reversible Protein Conjugation

Sugar-containing polymeric materials exhibit high affinities for proteins, hence sugar-conjugated block copolymers, star copolymers and polymer brushes have been investigated for use in biomedical applications.^63–65^ These thiol-reactive polymer brushes are unique platforms for the selective catch-and-release of proteins (Figure 6a). As a first proof of concept experiment, mannose functionalized brushes were used to demonstrate the selective binding and successive release of ConA from a mixture of lectins (Concanavalin A (ConA) and Peanut Agglutinin (PNA)). For these experiments, the copolymer brush P2 was first modified with a thiol-containing ligand, in particular, 2-mercaptoethyl alpha-D-mannopyranoside (mannose-SH), synthesized according to literature,^66,67^ via the thiol–disulfide exchange reaction. After the conjugation of mannose-thiol, the specific lectin binding efficiency of this surface was evaluated by immersing the mannosylated polymer brush P2 in a mixture of FITC-ConA (0.72 μM, 0.075 mg/mL) and RhB-PNA (0.68 μM, 0.075 mg/mL) in Mn^2+^ and Ca^2+^ containing HEPES buffer saline (10 mM) for 1h. A nonmannosylated P2 brush was used as a control and presented with the same protein solution. After 1 h, the surfaces were washed with HEPES buffer to remove unbound PNA, and lectin binding was investigated by using fluorescence microscopy. Green fluorescence showed successful immobilization of ConA lection onto the mannose-containing P2 brush due to ConA-mannose interaction efficiency (K_*d*_ = 2.89 × 10^–6^ M)^68^ (Figure 6b). On the other hand, there was no red fluorescence observed from Rhodamine-PNA, which suggests that PNA did not show binding toward the mannose-containing surface. The nonmannosylated P2 control brush did not show any fluorescence, which underlines the selective, mannose-binding mediated immobilization of ConA (see inset Figure 6b). As a consequence, this experiment demonstrates the ability of the mannose functional polymer brush to capture ConA from a mixture of lectins selectively. Finally, the selectively captured FITC-ConA was released by incubating the brush with a 10 mM DTT solution in DMSO for 24 h. The removal of the FITC-ConA intensity was investigated using fluorescence microscopy. Green fluorescence disappeared due to disulfide cleavage in the presence of DTT, suggesting the release of ConA protein (Figure 6c).

**Figure 6:**
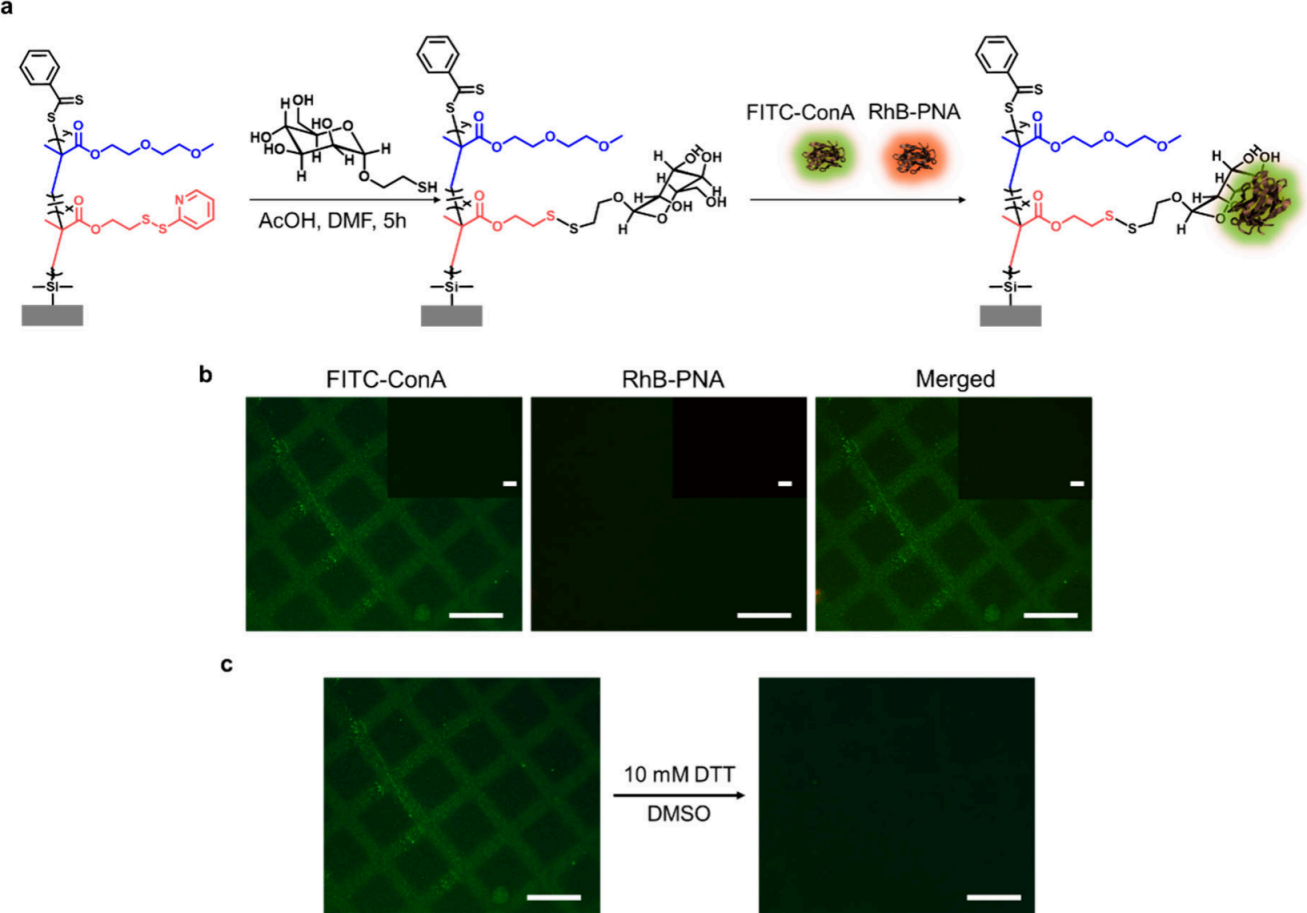
(a) Fabrication of a mannose-containing P2 brush for lectin binding affinity. (b) Fluorescence microscopy images of a mannose-containing P2 brush treated with FITC-ConA and RhB-PNA. (c) Fluorescence microscopy images of FITC-ConA immobilized P2 brush before and after DTT treatment. The scale bar is 100 μm.

### Fabrication of Cell-Attracting Polymer Brushes

After the reversible modification capabilities of PDS-containing polymer brushes with thiolated molecules were demonstrated, the efficiency of PDS-containing polymer brush surfaces to attach and release cells was investigated. PDS-containing P2 copolymer brushes were modified with the cell adhesive RGD tripeptide motif for these experiments (SI Figure S7a). The RGD peptide shows specific binding to the integrin receptors expressed on the surface of cells and thus promotes their attachment.^69^ To date, many examples of RGD-targeting group conjugated polymeric systems have been reported.^70–72^ To immobilize this peptide via disulfide exchange, a tetrapeptide incorporating a cysteine residue at the C-terminus was used. The sample substrate was immersed in a 1 mg/mL DMF solution of the peptide to modify the brush. The successful conjugation of the RGD peptide onto the polymer surface was established using FITC dye. The N-terminal amine group on the arginine residue of RGD peptide reacted with FITC, and a patterned green fluorescence was observed (SI Figure S7b). An unmodified P2 polymer brush was utilized as a control surface, and as expected, there was no green fluorescence upon treatment with FITC dye due to the lack of an amino group (SI Figure S7c).

In recent years, harvesting of specific surface-bound cells with high viability has been investigated for biomedical applications.^73–75^ For example, Wang and co-workers demonstrated endonuclease-responsive aptamer-functionalized hydrogel coatings for the catch and release of cancer cells.^73^ Minko and co-workers reported an RGD-peptide mediated cell capturing and enzyme-free cell harvesting method from thermoresponsive polymer surface.^74^ Herein, we explored whether our platform could provide a cell-harvesting system, where specific cells bound to the surface could be effectively collected under mild conditions. To this end, L929 mouse fibroblast cells grown in DMEM at 37 °C under a 5% CO_2_ atmosphere were seeded on the RGD-modified P2 polymer brush surfaces to probe cell attachment on the peptide-modified surface (Figure 7a). Unmodified P2 polymer brushes and P1 P­(DEGMA) brushes were used as control surfaces. The effect of RGD conjugation on cell attachment was compared in the presence and absence of the RGD peptide. After 24 h of incubation, the actin filaments and nuclei of cells were stained with Alexa Fluor-phalloidin 488 and DAPI, respectively, and analyzed by using fluorescence microscopy. A clear difference was observed between the RGD-modified P2 brush and the control P1 P­(DEGMA) brush. Figure 7b shows that the control P1 brush is relatively bioinert due to the antibiofouling nature of the oligoethylene glycol groups (10 cells/mm^2^). In contrast, as seen in Figure 7d, modifying the P2 brush with the cell adhesive RGD peptide promoted cell attachment (295 cells/mm^2^). Interestingly, although significantly less than that of the RGD-modified surface, some cell attachment was also observed on the PDS functionalized P2 polymer brush surfaces (47 cells/mm^2^) (Figure 7c and e), which could be due to the interaction of thiol groups on cell surface proteins with the thiol-reactive PDS groups.

**Figure 7:**
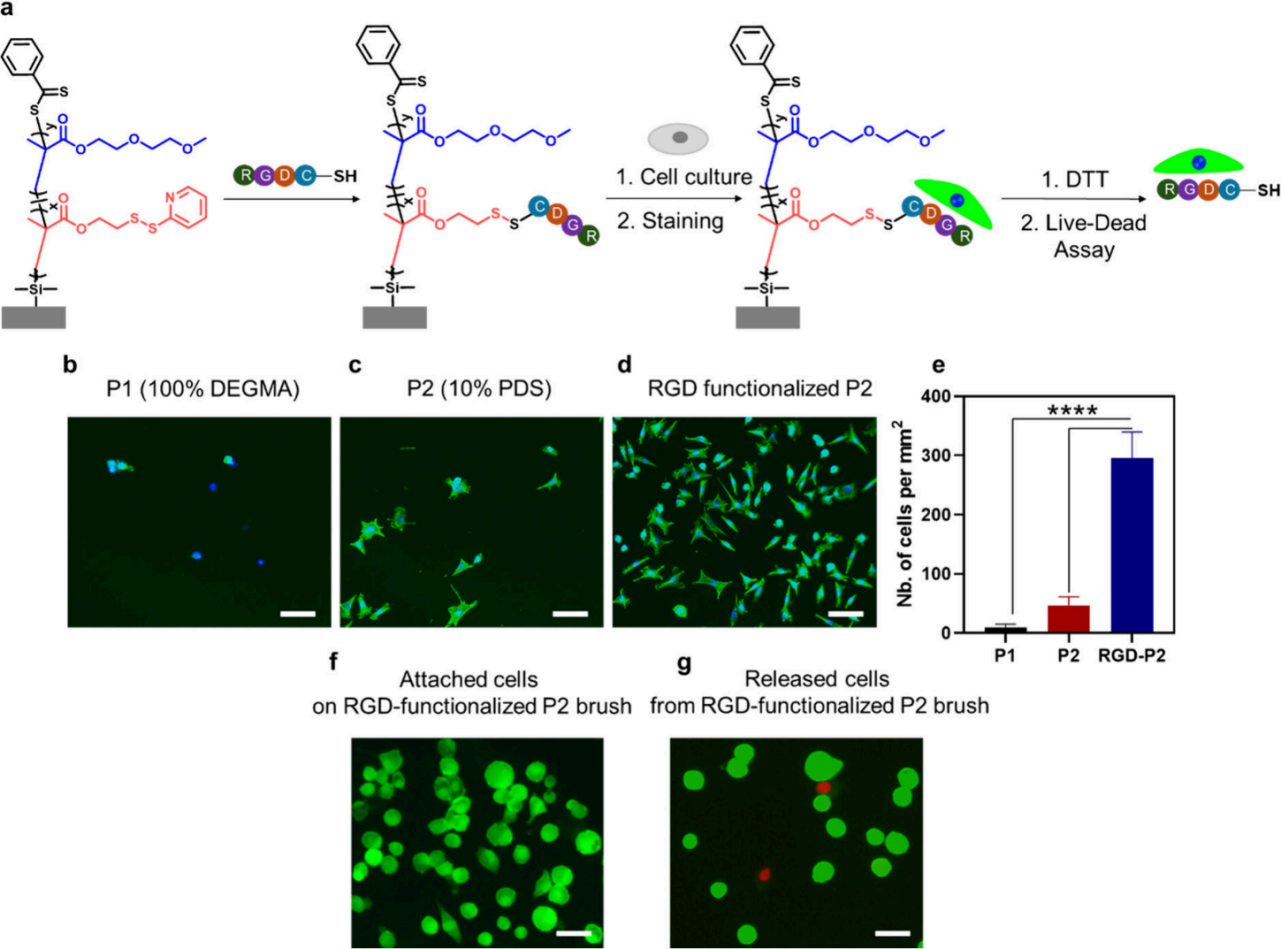
(a) Fabrication of an RGD-containing polymer brush for cell catch and illustration of cell release from release. (b) Merged fluorescence microscopy images of cells immobilized on (b) the P1 surface, (c) the P2 surface, and (d) RGDC-conjugated P2 surfaces after actin filament staining with Alexa Fluor 488 and nuclei staining with DAPI. The scale bar is 100 μm (e). The number of cells attached onto polymer brush surfaces was 1 mm^2^. Values are the average of four images taken with a 10× objective. Asterisks indicate statistically significant differences between the indicated samples ****p < 0.0001). (f) Fluorescence microscopy image of calcein-AM and PI-stained cells onto the RGD peptide-conjugated brush surface. (g) After detachment from the polymer surface, the fluorescence microscopy image of calcein-AM and PI-stained cells. The scale bar is 50 μm.

After the successful attachment of cells onto the peptide conjugated surface, cell release from the surface was assessed without compromising their viabilities. A live–dead assay was used to monitor cell viability after detachment from the surface. L929 mouse fibroblast cells were seeded onto an RGD-conjugated polymer brush surface and incubated for 24 h. After incubation, surface-attached cells were stained with calcein-AM and propidium iodide (PI), and the cells were visualized under a fluorescence microscope. The green fluorescence and cell morphology indicated their viable nature (Figure 7f). L929 cells were seeded onto an RGD-modified surface for cell release and the viability assay. After 24 h of incubation, surface attached cells were exposed to a 5 mM DTT solution. The cells detached from the surface due to breaking redox-responsive disulfide bonds with DTT. After transferring them into a well plate, the surface detached cells were collected and stained with calcein-AM and propidium iodide. In this assay, calcein-AM showed esterase activity in viable cells and gave a green fluorescence. Propidium iodide demonstrated binding to the DNA of dead cells and gave a red fluorescence. Fluorescence microscopy analysis displayed that the viability of cells was not compromised after release from the polymer brush surface. (Figure 7g).

### Orthogonal Functionalization for Localized Delivery to Cells

We envisioned that such an orthogonally functionalizable platform, where one conjugation is reversible, and the other nonreversible, may be employed to fabricate a platform that is able to attract specific cells and then kill them locally (Figure 8). To this end, we conjugated cell-adhesive peptides on the surface of the polymer brushes and attached cytotoxic anticancer drugs using the redox-responsive, reversible disulfide linker. To fabricate these surfaces, the PDS-functionalized copolymer brush P2 was treated with 3-aminopropanol, followed by immersion in a DMF solution containing 2 mg/mL of a maleimide-functionalized cyclic RGD peptide. To investigate the efficiency of cRGD-Mal conjugation, the thiol content of the thiol-terminated P2 brush was determined using Ellman’s assay before and after RGD conjugation. The remaining thiol content on the polymer surface after peptide attachment was estimated as 3%, which suggests that the efficiency of maleimide-containing cRGD peptide conjugation was 97%. Another experiment to assess the peptide conjugation efficiency was conducted using the TAMRA-Mal dye. After conjugation of cRGD, the surface was treated with TAMRA-Mal for 3h, to investigate the presence of unreacted thiol groups. Lack of significant red fluorescence on the surface also suggests that the cRGD peptide was conjugated to thiol end groups on the brush surface with high efficiency (SI Figure S8).

**Figure 8:**
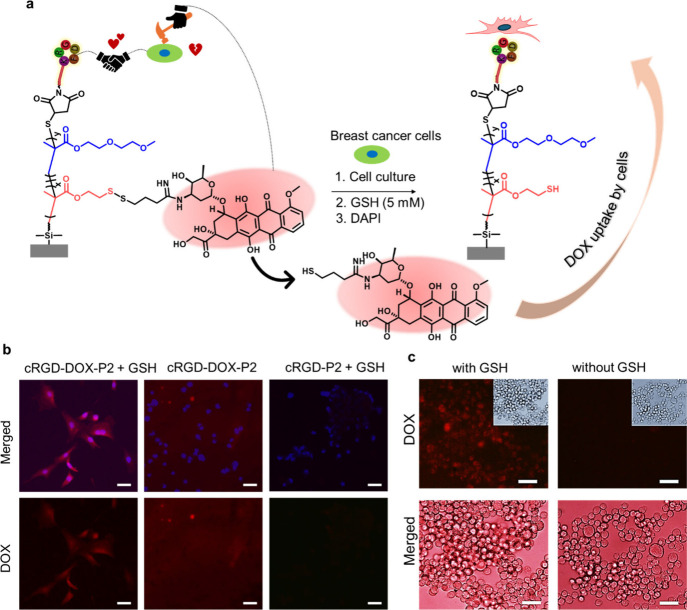
(a) Schematic illustration of the DOX release and internalization efficiency in the presence and absence of GSH. (b) Fluorescence merged, and DOX channel images of MDA-MB-231 cells attached surfaces with and without GSH treatment and illustrated DOX internalization efficiency by the cells. (c) Fluorescence and bright field images of detached cells from the surface treated with GSH and cell media. The scale bar is 50 μm.

As a control, another thiol-containing surface was obtained after 3-aminopropanol treatment with a 3-hydroxypropyl maleimide solution (1 mg/0.5 mL in DMF). Mouse fibroblasts (L929) were seeded onto cRGD- and hydroxypropyl-containing P2 control surfaces. After 24 h of incubation, the actin filaments and nuclei of the cells were stained with Alexa Fluor 488 and DAPI, respectively, and the surfaces were visualized under a fluorescence microscope. As expected, enhanced cell immobilization and proliferation were observed on the cRGD-functionalized P2 brush (130 cells/mm^2^) (SI Figure S9). In contrast, cell attachment and spreading remained significantly lower on the hydroxypropyl-functionalized P2 brush (41 cells/mm^2^).

As the next step, doxorubicin, an anticancer drug, was conjugated to the side chains of the polymer brush. To this end, polymer brush P2 was treated with DOX-SH solution in the presence of a catalytic amount of acetic acid. As a control, the P2 surface was incubated with a nonthiol functional DOX solution under the same reaction conditions. The characterization of the surfaces was performed with fluorescence microscopy analysis. Red fluorescence suggested DOX-SH conjugation to the P2 brush via the thiol–disulfide exchange reaction (SI Figure S10b). On the other hand, the fluorescence image of the control group did not give red fluorescence because of the lack of any thiol group in the unmodified drug (SI Figure S10b, inset).

After individually establishing the successful attachment of cell-adhesive peptide and the anticancer drug, the next step was to evaluate the efficacy of the combined system, i.e., if the drug and peptide-conjugated surfaces are feasible for capturing and killing cancer cells (Figure 8a). To this end, PDS groups of the P2 polymer brush were functionalized with DOX-SH, and cRGD peptide was conjugated to the chain ends of the surface-grafted P2 brush. After obtaining the DOX and RGD-conjugated P2 brush, MDA-MB-231 breast cancer cells were seeded on this surface. As a control, only the cRGD-conjugated P2 surface was used as the control surface without modifying with DOX-SH. After 24h incubation, the surfaces were incubated with GSH (5 mM) for 5h. The cRGD and DOX-containing polymer brush P2 was used as another critical control experiment in which the surface was not incubated with GSH. After 5h, cells on surfaces were stained with DAPI, and cells were visualized using fluorescence microscopy. MDA-MB-231 cells on cRGD and DOX-conjugated P2 brush surface (cRGD-DOX-P2) gave red fluorescence when the cRGD-DOX-P2 surface was treated with a GSH solution. This image revealed that the cRGD-DOX-P2 surface demonstrated DOX release due to breaking of the disulfide bonds in the presence of GSH, which was then internalized by the cells (Figure 8b). Furthermore, MDA-MB-231 cells on the cRGD-DOX-P2 surface did not display red fluorescence because this surface was not incubated with GSH. The surface remained red, as expected, indicating that the DOX remains bound to the surface.

To clarify whether DOX was internalized by these cells, a control experiment was conducted. MDA-MB-231 cells were seeded on the cRGD-DOX-P2 surfaces. After being incubated for 24 h, one of these surfaces was treated with GSH for 5 h. Another surface was treated with only cell media. After incubation, these surfaces were exposed to trypsin, detached cells were collected after the centrifugation step, and cells were visualized under the fluorescence microscope. Fluorescence images are given in Figure 8c. The fluorescence images were overlapped with bright field images. The cells showed red fluorescence due to DOX internalization after trypsinization of the GSH-treated surface. On the other hand, detached cells from the GSH-untreated surface did not reveal any significant red fluorescence. Importantly, this result demonstrated DOX release and internalization in the presence of GSH. After that, detached cells from GSH-treated and GSH-untreated surfaces were seeded into a well-plate in the same numbers. The cells were incubated at 37 °C for 24 h, and then, the cells were washed with 1xPBS. After washing, cells were analyzed under an inverted microscope. While detached cells from the GSH-untreated surface showed good morphology with an elongated shape, detached cells from the GSH-treated surface demonstrated spherical and distorted morphology. In addition to morphology, detached cells from the GSH-untreated surface displayed more attachment to the well plate than that of the GSH-treated surface (SI Figure S11). Moreover, the proliferation efficiency of MDA-MB-231 cells on cRGD-DOX-P2 surfaces without and with GSH (5 mM) treatment was examined for 48 h (SI Figure S12). After staining protocol with AF488 and DAPI, surfaces were visualized under the fluorescence microscope. The cells demonstrated the expected attachment and proliferation for the ones on the cRGD-DOX-P2 surface without adding GSH. On the other hand, the number of proliferated cells from the cells on the GSH treated cRGD-DOX-P2 surface decreased in time, since they had internalized the DOX released upon treatment with GSH.

## Conclusions

In this work, redox-responsive PDS-containing polymer brushes were fabricated with a tunable PDS ratio via SI-RAFT polymerization. Obtained thiol-reactive polymer brushes allow postpolymerization modification with thiol-containing molecules via the thiol–disulfide exchange reaction. Selective functionalization with thiol-containing molecules was demonstrated using a fluorine-tagged benzyl thiol, a thiol-containing mannose sugar, a thiol-containing fluorescent dye, and a drug. In addition, the cell adhesive RGD peptide was conjugated to polymer brushes to enable cellular attachment, and they could be released in the presence of a thiol-based reducing agent to harvest them with high viability. Moreover, reducing the number of thioester chain end groups paves the way for the secondary functionalization of polymer brushes. RAFT groups were reduced to thiol units and conjugated with a maleimide-containing fluorescent dye and cell adhesive peptide via thiol-maleimide chemistry. This orthogonally functionalizable platform was utilized to fabricate an interface for a catch-and-kill device. In particular, an anticancer drug was conjugated to the side chains of the polymer brush using the thiol–disulfide exchange reaction; cell adhesive cyclic RGD peptide was conjugated to the top of polymer brush chains using the Michael type thiol-maleimide addition reaction. Thus, the modified surface was able to attach cancer cells, which could be killed by the local release of the disulfide-linked drug. Notably, the redox-responsive polymer brush interface disclosed here would be an attractive platform for various biomedical applications entailing capture-and-release protocols and the realization of devices for the targeted capture of disease cells and their localized eradication.

## Supplementary Material



## Notes

Experimental section, additional, AFM, XPS spectra, fluorescence microscope images and additional *in vitro* cell data (PDF)
