# Supplementary material for: Orthogonally Functionalizable Redox-Responsive Polymer Brushes: Catch and Release Platform for Proteins and Cells
Source: J Am Chem Soc. 2025 Jul 3;147(28):24672–83. doi: 10.1021/jacs.5c05856 (PMC12272546; doi:10.1021/jacs.5c05856)
Supplement: Supplementary file 1 [file ja5c05856_si_001.pdf]

Supporting Information

Orthogonally Functionalizable Redox-Responsive Polymer Brushes: Catch and Release Platform for Proteins and Cells

Aysun Degirmenci,[a] Rana Sanyal,[a,b] Harm-Anton Klok,[c] Amitav Sanyal[a,b]*

[a] Department of Chemistry, Bogazici University, Bebek, Istanbul, 34342, Türkiye

[b] Center for Targeted Therapy Technologies, Bogazici University, Istanbul, 34684, Türkiye

[c] Institut des Materiaux and Institut des Sciences et Ingenierie Chimiques, Laboratoire des Polymeres, Ecole Polytechnique Federale de Lausanne (EPFL), Batiment MXD, Station 12, Lausanne CH-1015, Switzerland.

Email: amitav.sanyal@bogazici.edu.tr

Table of Contents

Experimental Section 3

Additional *In vitro* Cell Data 22

References 24

Experimental Section

Materials. Di(ethylene glycol) methyl ether methacrylate (DEGMA) was obtained from Sigma-Aldrich and filtered through the basic aluminum oxide to remove the inhibitor before use. AIBN was purchased from Sigma Aldrich and was used after recrystallization from methanol. 2-Mercaptoethanol was purchased from Merck. 2,2'-Dipyridyl disulfide was obtained from IS Chemical. The linear arginine-glycine-aspartate-cysteine (RGDC, RGD-SH) peptide, 4′,6-diamidino-2-phenylindole (DAPI), 4-trifluoromethylbenzyl mercaptan (TFBM), tetramethylrhodamine-5-maleimide (TAMRA) and Ellman's reagent (5,5′-dithio-bis-(2-nitrobenzoic acid, DTNB) were obtained from Sigma Aldrich. Alexa Fluor 488 Phalloidin was obtained from Invitrogen. A live-dead assay kit consisting of calcein-AM (live) and propidium iodide (dead) was purchased from Sigma Aldrich. FITC-ConcanavalinA (104 000 g/mol) and Rhodamine-PNA (110 000 g/mol) were purchased from Molecular Probes and Vector. Pyridyl disulfide alcohol (PDS-OH),[1] pyridyl disulfide methacrylate (PDSMA),[1] and thiol-containing mannose[2,3] were synthesized following previously described procedures. Thiol-bearing BODIPY dye (BODIPY-SH)[4] and DOX-SH[5] were synthesized using published methods. Surface attachable RAFT agent and RAFT agent-coated silicon surfaces (Si-RAFT-CTA) were prepared according to a literature procedure.[6] Si/SiO_2 wafer pieces (1 cm × 1 cm) were used in the experiments.

Methods. ^1H NMR spectrum of PDSMA was recorded in $CDCl_3$ on Bruker Avance Ultrashield 400 (400 MHz). Surface characterization was done using X-ray photoelectron spectroscopy (XPS) (K-Alpha, Thermo Scientific). XPS data analysis was performed using CasaXPS processing software. All XPS spectra were calibrated on the aliphatic carbon signal at 285.0 eV. The incorporation of PDSMA into the polymer brushes was evident from the nitrogen signal and PDSMA/DEGMA ratio was calculated using N/O ratio. Nicolet 380 (Thermo Fisher Scientific, Inc.) instrument equipped with a Harrick Scientific GATR accessory, and a Ge crystal was used for FT-IR analysis. Polymer brush thicknesses were measured using a Nanosurf AFM. 2D and 3D AFM images of patterned brush were processed using Gwyddion software. In AFM thickness measurements, NCLR-10 type cantilever was utilized. Typical technical data are given. (Thickness: 7 μm/ Length: 225 μm/ Width: 38 µm, Resonance Frequency: 190 kHz and Force Constant: 48 N/m). During AFM analysis, scan size was chosen as 50 μm. Time/line and sample/line are 1.2 sec and 256, respectively. The scan angle is 0°. Fluorescence microscopy was

performed using LD-A-Plan 10x/0.30 objective in a Zeiss Axio Observer inverted microscope (ZEISS Fluorescence Microscopy, Carl Zeiss Canada Ltd., Canada). Cell densities on polymer brushes were quantified using Image J software. Statistical analysis was performed using one way ANOVA test in GraphPad Prism software.

Immobilization of surface RAFT agent. The synthesis of surface RAFT agent and immobilization of Si/SiO_2 surfaces with RAFT agent were undertaken according to a literature procedure.[6] Briefly, Si/SiO_2 surfaces were washed with acetone and ethanol by sonication for 10 min and dried under a nitrogen flow. After that, the silicon surfaces were cleaned using a Novascan PSD Series UV/Digital Ozone System for 30 min. Subsequently, the cleaned silicon wafers were immersed in RAFT agent solution in anhydrous toluene (1 mM) and incubated for 6 h. After that, silicon surfaces were washed with excess toluene and CH_2Cl_2 and dried with a nitrogen flow.

Preparation of patterned RAFT-agent immobilized surface

RAFT chain transfer agent-immobilized silicon surfaces were exposed to high-intensity UV irradiation through a photomask for 10 min. to generate patterned polymer brushes.

Surface polymerization of DEGMA (P1 DEGMA homopolymer brush 100/0). Di(ethylene glycol) methyl ether methacrylate (DEGMA, 1.5 g, 8 mmol) and AIBN (2.16 mg, 0.013 mmol) were dissolved in anhydrous DMF and degassed with N_2 for 30 min. In a separate vial, the RAFT agent-coated wafers were purged with N_2 for 15 min, and subsequently, wafers were treated with the solution of DEGMA/AIBN under nitrogen at 75 ^oC for 5 h. At the end of 5 h, silicon wafers were washed with DMF and CH_2Cl_2. They were dried under a flow of nitrogen.

Fabrication of copolymer brushes. A typical procedure is outlined for synthesizing polymer brush P2 (90/10). PDSMA (100 mg, 0.39 mmol), DEGMA (660 mg, 3.51 mmol), and AIBN (1.28 mg, 7.8 x 10^{-3} mmol) were dissolved in anhydrous DMF (1.5 mL). The solution was degassed by using nitrogen gas bubbling for 30 min. The surface-attachable RAFT-CTA modified Si/SiO_2 surface (Si-RAFT-CTA) was put into a vial and purged with N_2 for 15 min. The monomer solution was transferred to the surface, including the vial under N_2, and this vial was heated at 75 ^oC for 5

h. After that, the solution was removed, and the coated surface was washed with DMF and CH_2Cl_2 and dried using a nitrogen stream. Polymer brushes P3, P4, and P5 were also obtained using the same method, which changed the feed ratio of monomers.

Fabrication of homopolymer brush P5 (0/100) (PDSMA homopolymer brush). PDSMA (100 mg, 0.39 mmol) and AIBN (0.128 mg, 7.8 x 10^{-4} mmol) were dissolved in anhydrous DMF (0.5 mL). The solution was degassed by using nitrogen gas bubbling for 30 minutes. The surface-attachable RAFT-CTA modified Si/SiO_2 surface (Si-RAFT-CTA) was put into a vial and purged with N_2 for 15 minutes. The monomer solution was transferred to the surface, including the vial under N_2, and this vial was heated at 75 °C for 5 h. After that, the solution was removed, and the coated surface was washed with DMF and CH_2Cl_2 and dried using a nitrogen stream.

Functionalization of the polymer brush with BODIPY-SH dye. A BODIPY-SH solution (1 mg/mL) was prepared in anhydrous DMF, and a catalytic amount of acetic acid (CH_3COOH) was added. Copolymer brush P2 was treated with this solution for 5 h. After that, the surface was washed with DMF and THF and dried under a stream of nitrogen.

Cleavage of dyes from functionalized polymer brush. BODIPY-conjugated polymer brush P2 was treated with 10 mM DTT solution in PBS: DMSO (90:10) for 24 h. The fluorescence microscopy images were recorded during the predetermined time interval.

Functionalization of the polymer brush with TFBM. Polymer brush P2 was treated with a TFBM solution (10 mg/mL in DMF) containing acetic acid (52 μL) for 24 h. Afterward, the surface was washed with a gentle amount of DMF and THF and dried under a nitrogen flow.

Functionalization of the polymer brushes with mannose-SH for protein immobilization. Polymer brush P2 was treated with mannose-SH solution (10 mg/mL in DMF) containing acetic acid (40 μL) for 5 h. After that, the surface was washed with DMF and THF and dried under a stream of nitrogen. Then, the mannose-containing surface was incubated with a mixture of FITC-ConA (0.72 μM, 0.075 mg/mL) and Rhodamine-PNA (0.68 μM, 0.075 mg/mL) in 20 mM HEPES

(1.0 mM $MnCl_2$, 1.0 mM $CaCl_2$, 0.15 M NaCl, adjusted to pH 7.4) for 60 min. Subsequently, the surface was washed with 20 mM HEPES solution and dried under a nitrogen flow.

Cleavage of FITC-ConA from functionalized polymer brush. FITC-ConA conjugated mannosylated polymer brush P2 was treated with 10 mM DTT solution in DMSO for 24 h. The fluorescence microscopy images were recorded during the predetermined time interval.

Functionalization of the polymer brush with thiol-containing peptide. Linear RGD-SH was dissolved in anhydrous DMF (1 mg/mL), and the catalytic amount of acetic acid (50 μL) was added. Polymer brushes (P2) were incubated with this solution for 4 h. RGD-modified brushes were washed with a gentle amount of DMF and THF and dried using a nitrogen stream.

FITC conjugation on peptide-conjugated polymer brushes. Fluorescein isothiocyanate (FITC) (1 mg, 0.0026 mmol) was dissolved in anhydrous DMF, and triethylamine (8.4 μL, 0.06 mmol) was added. RGD functionalized P2 polymer surface was immersed into this solution and incubated for 4 h. As a control experiment, the RGD unmodified P2 polymer brush was treated with FITC solution under the same conditions. Subsequently, dye-conjugated, and control surfaces were washed with a gentle amount of DMF and THF and dried under a nitrogen stream.

Cell attachment on peptide-conjugated polymer brush. L929 mouse fibroblast cells were used, which were grown in a 5% CO_2-containing atmosphere at 37 °C. Cells were cultured in Dulbecco's Modified Eagle's Medium (DMEM) media supplemented with 10% fetal bovine serum (FBS) (PAN Biotech). L929 mouse fibroblasts (15000 cells) were seeded onto prepared RGD-SH functionalized P2 surfaces. P2 polymer brush and bare DEGMA polymer brush P1 were used as control surfaces. After dropping the cell suspensions onto polymer brush surfaces, they were incubated at 37 °C in a 5% CO_2-containing atmosphere for 2 h. Afterward, cell media (1 mL) was added to each well-containing polymer brush surface. After incubation (24 h), cell media was removed, polymer interfaces were rinsed with 1 x PBS (3 times), and the cells were fixed with 3.7 % formaldehyde solution for 10 min at 37 ºC. For staining filamentous actins (F-actins), cells were incubated with 0.1% Triton X-100 in PBS for 5 min. Then polymer brush surfaces were washed

with 1 x PBS (1 mL x 3 times), and they were incubated in Alexa Fluor 488 phalloidin solution (5 units/mL concentration containing 1% bovine serum albumin (BSA) in 1 x PBS) for 20 min at 37°C. After that, polymer brush surfaces were washed with 1 x PBS (1 mL x 3 times) to remove the dye. Finally, cell nuclei were stained with DAPI for 15 min at room temperature, and polymer brush surfaces were washed with 1 x PBS (1 mL x 3 times). The resulting images of cells were taken via Zeiss AxioVision and processed via Zen Lite software.

Cell detachment from peptide-conjugated brush and viability assay. L929 fibroblast cells (45000 cells/350μL) were seeded on the RGD functionalized polymer brush surfaces and incubated at 37 °C with 5% CO_2 for 3 h. After 3 h, DMEM (1 mL) was added to each surface, and incubation was continued for 24 h. After that, cell-attached surfaces were incubated with 5 mM DTT solution for 25 min, and released cells from polymer surfaces were washed with 1 x PBS (1 mL x 3 times) and stained via a live/dead assay kit. Solution A (calcein-AM, 10 μL) and solution B (propidium iodide, 5 μL) were diluted with 5 mL 1 x PBS to prepare the assay solution. Cells were incubated with this assay solution at 37 °C with 5% CO_2 for 25 min. Subsequently, stained cells were washed with 1 x PBS (1 mL x 3 times). Live/dead cell viability was visualized by fluorescence microscopy and processed via Zen Lite software.

Functionalization of the polymer brush with thiol-containing doxorubicin. Polymer brush P2 was treated with thiol-containing doxorubicin (DOX-SH, 1 mg/0.2 mL in DMF) containing acetic acid (3 μL) for 6 h. After that, the surface was washed with DMF and THF and dried under a stream of nitrogen. As the control experiment, DOX was used.

Orthogonal functionalization with aminopropanol and maleimide-containing molecules and cell adhesive peptide. Copolymer brush P2 was treated with aminopropanol (0.0576 mmol, 4.4 μL) in DMF (0.5 mL) in the presence of triethylamine (0.0576 mmol, 8.4 μL) for 20 h. After the washing step, a solution of tetramethylrhodamine maleimide (TAMRA-Mal) (30 μL from 1 mg/mL in THF) was dropped onto this surface and incubated for 3 h. Then, the surface was washed with THF. As control experiment, we conducted a control experiment with Rhodamine B (RhB) dye. Thiol-containing P2 brush generated by reducing phenyl dithioester groups was treated with Rhodamine B dye (1 mg/mL in THF) for 3h. After washing with excess amount of THF, the

surface was analyzed under fluorescence microscopy analysis. The reaction efficiency was determined by Ellman's assay. Thiol-containing P2 brush was treated with Ellman's reagent (5,5′-dithio-bis-(2-nitrobenzoic acid, DTNB) (20 μL from 2 mg/mL 0.1M sodium phosphate, pH 8.0, containing 1mM EDTA) for 1h before and after conjugation reaction with TAMRA-Mal. After 1h treatment, the UV of solution was measured by Nanodrop to determine absorbance of highly chromogenic 5-nitro-2- thiobenzoic acid (TNB) at 412 nm. After drying, this surface was incubated with a BODIPY-SH solution (30 μL from 1 mg/mL in DMF) containing the catalytic amount of acetic acid (CH_3COOH) for 3 h. At the end of 3 h, the polymer surface was washed with DMF and THF and dried under a stream of nitrogen.

Another functionalization was done using N-(3-hydroxypropyl) maleimide. The RAFT end group containing the PDS polymer brush was treated with 3-amino-1-propanol for this. Then, this surface was incubated with the solution of N-(3-hydroxypropyl) maleimide (40 μL, 1 mg/0.5 mL) in DMF for 5h.

For cell adhesive cyclic RGD functionalization, the thiol end group containing surface, which was immobilized with 3-amino-1-propanol, was treated with maleimide-containing cyclic RGD peptide (cRGD-Mal) solution (1 mg/0.5mL) in DMF for 5 h. After the conjugation reaction, the surface was washed with THF and dried under a nitrogen flow. To check efficiency of cRGD-Mal conjugation, we conducted Ellman's assay. Thiol-containing surface, generated by reducing the phenyl dithioester groups, were divided into two pieces. One piece was treated with Ellman's reagent (5,5′-dithio-bis-(2-nitrobenzoic acid, DTNB) (20 μL from 2 mg/mL) for 1h. After 1h treatment, the UV-Vis spectrum of solution was measured using a Nanodrop instrument to determine absorbance of highly chromogenic 5-nitro-2- thiobenzoic acid (TNB) at 412 nm. The other surface piece was incubated with maleimide-containing cRGD peptide for 5 h. After 5h, the surface was washed and Ellman's assay was performed to determine the unreacted thiol groups. To show efficiency of reaction was also done control experiment. Thiol-containing P2 brush after reducing dithioester group was divided to two parts. While one part was treated with maleimide-containing rhodamine dye (TAMRA-Mal) for 3 h, the other part was incubated with maleimide-functionalized cRGD peptide for 5 h. After conjugation of cRGD, the surface was treated with TAMRA-Mal for 3h to investigate the presence of unreacted thiol groups. Fluorescence microscope images were compared.

Fabrication of both DOX and cRGD-containing polymer brush. Polymer brush P2 was treated with aminopropanol and was immobilized with cRGD-Mal peptide for 5h. After 5h, the surface was washed with THF, and subsequently, it was conjugated with DOX-SH in the presence of triethylamine for 3 h. At the end of 3 h, the surface was washed with THF and dried with a nitrogen flow.

Cell culture on the cRGD end group functionalized polymer brushes. For the actin staining experiment, L929 mouse fibroblast cells (20000 cells) were seeded on the cRGD end group functionalized PDS brush and incubated at 37 ^{o}C under a 5% CO_2 atmosphere for 24 h. N-(3-hydroxypropyl) maleimide functionalized PDS brush was used as a control surface. After 24 h, surfaces were washed with 1 x PBS (1 mL x 2 times). The cells were fixed with a 3.7 % formaldehyde solution for 10 min. Then, surfaces were cleaned with 1xPBS (1 mL x 2 times). The surfaces were incubated with 0.1% Triton X-100 solution for 5 min. After the washing step, surfaces were incubated in Alexa Fluor 488 phalloidin (AF488) solution (5 units/mL concentration containing 1% bovine serum albumin (BSA) in 1 x PBS) for 20 min at 37°C. DAPI stained cell nuclei after the washing step. The resulting images of cells were taken via Zeiss AxioVision and processed via Zen Lite software. MDA-MB-231 human breast cancer cells purchased from ATCC were used for the DOX release and internalization experiment. The cells were grown in a DMEM medium at 37 ^{o}C under a CO_2 atmosphere.

***In vitro* DOX release and cell internalization.** MDA-MB-231 cells (20 000 cells) were seeded onto cRGD and DOX-containing polymer brush surfaces and incubated for 24 h. Only the cRGD containing the PDS brush surface was used as the control surface. After 24 h incubation, these surfaces were treated with GSH solution (5 mM) for 5 h. Then, the GSH solution was removed, surfaces and cells were washed with 1 x PBS (1 mL x 2 times), and cells were fixed with 3.7% formaldehyde solution for 10 min at 37 ^{o}C. After washing with PBS, the cell nuclei were stained by DAPI. After staining, cells were washed with PBS, and the resulting images of cells were taken via Zeiss AxioVision and processed via Zen Lite software. For the control experiment, after cell-attached surfaces were treated with GSH and cell media for 5 h, surfaces were exposed to trypsin, and detached cells were collected and visualized under the fluorescence microscope.

Cell proliferation experiment on cRGD-DOX-P2 surface. MDA-MB-231 cells (20 000 cells) were seeded on the cRGD-DOX-P2 surface and incubated for 24 h. After incubation for 24 h, GSH (5 mM) was added. Then, the cells attached to the surfaces were treated with GSH for 48 h. As a control experiment, surface-attached cells were treated with only cell media. At 0 h (GSH addition day), 24 h, and 48 h, F-actins and nuclei of attached cells were stained by AF488 and DAPI, respectively. Cells were visualized under the fluorescence microscope.

Table S1. The properties of polymer brush (P1-P5) and theoretical and calculated atomic ratios.

Brush type	Theoretical Atomic Ratio%				Experimental Atomic Ratio % by XPS			
	C%	O%	N%	S%	C%	O%	N%	S%
**P1**	70.0	30.0	-	-	74.23	25.77	-	-
**P2**	69.17	28.58	0.75	1.5	71.28	26.69	0.51	1.52
**P3**	69.11	26.48	1.47	2.94	70.06	25.27	1.05	3.62
**P4**	69.06	24.46	2.16	4.32	70.68	24.41	1.36	3.55
**P5**[a]	68.75	12.5	6.25	12.5	36.71	23.33	2.00	10.88

[a]Si peaks were observed at 27.08 due to low grafting density.

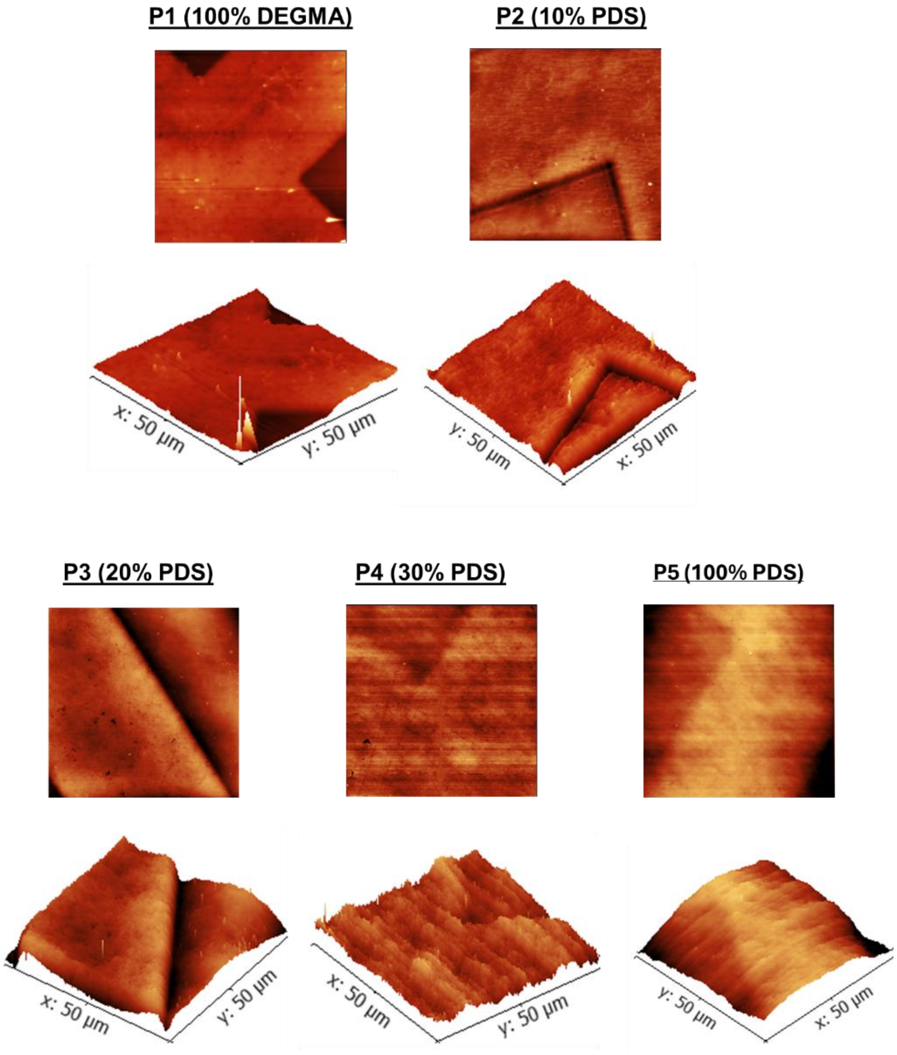

Figure S1. 2D and 3D AFM images of patterned P1-P5 polymer brushes.

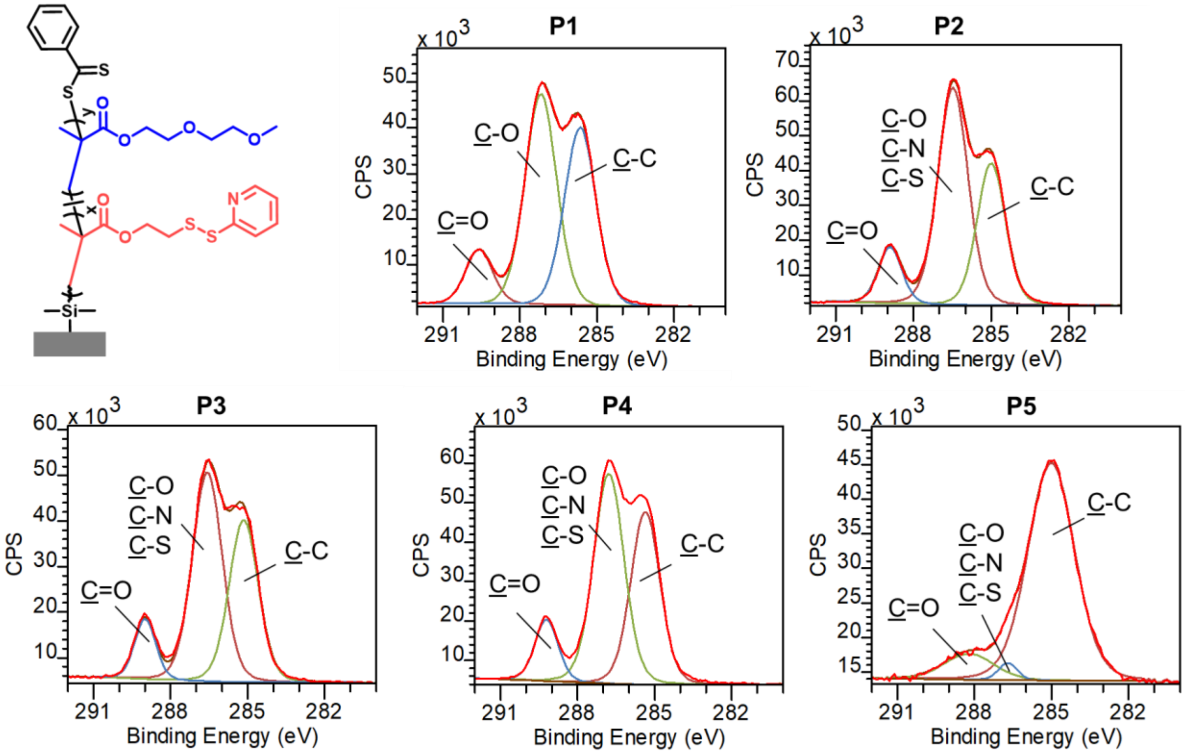

Figure S2. High-resolution C1s XPS spectra of polymer brushes P1-P5.

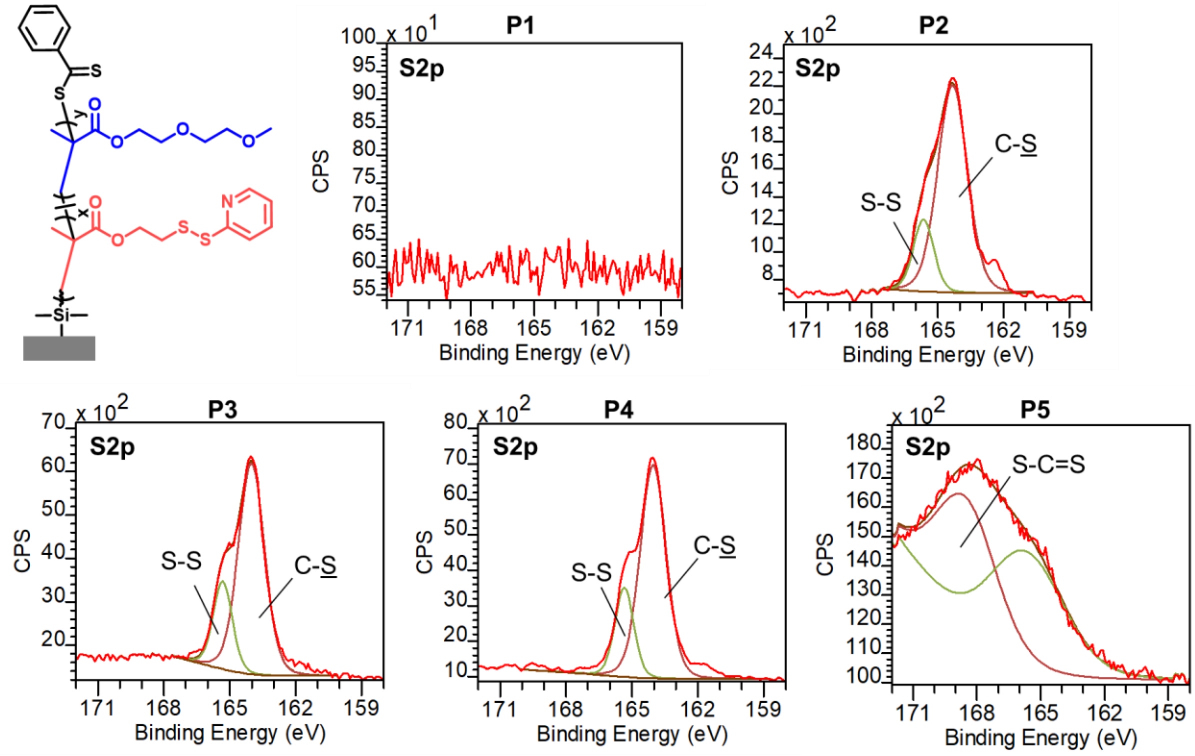

Figure S3. High-resolution S2p spectra of polymer brushes P1-P5.

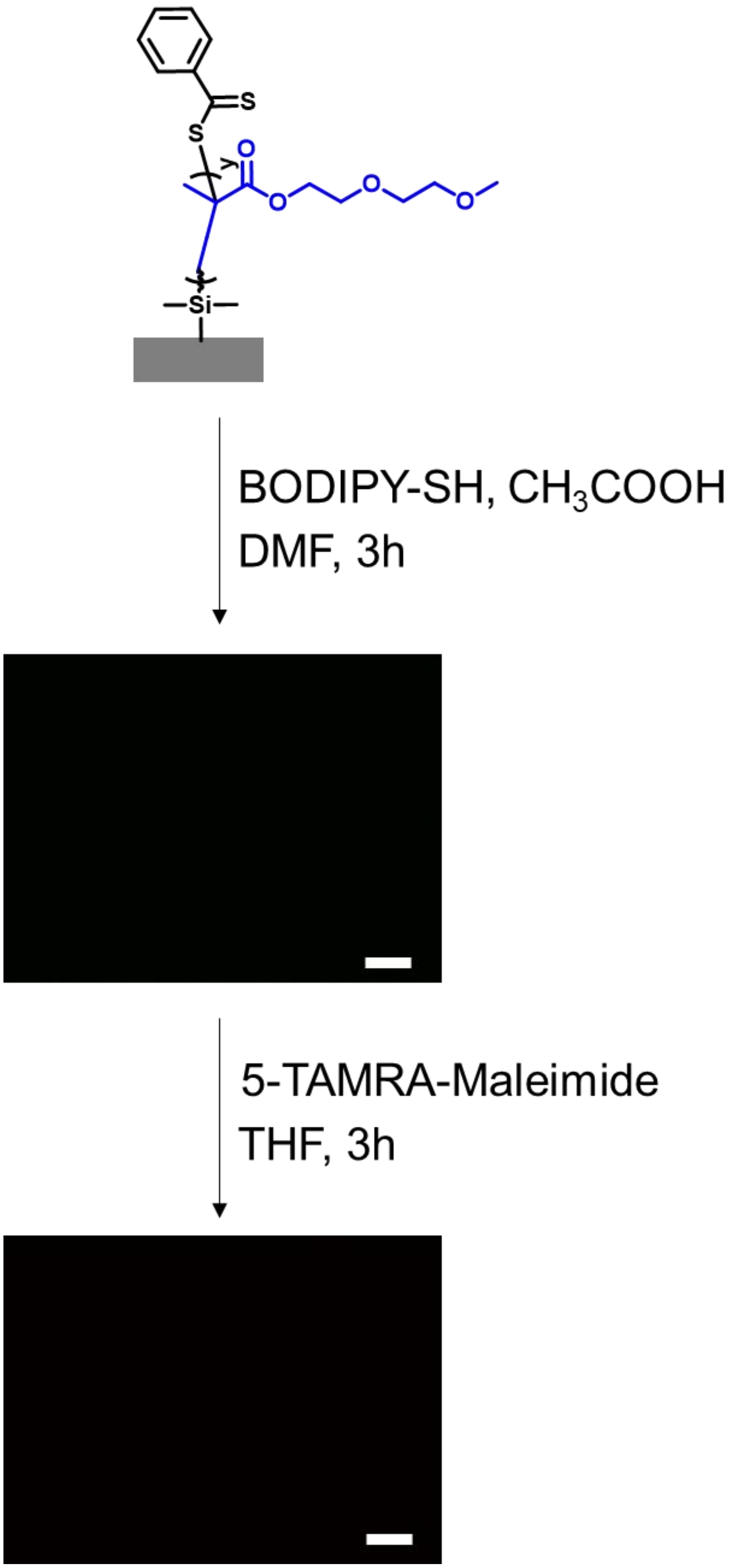

Figure S4. The possibility of the reaction of BODIPY-SH with the polymer chain-end.

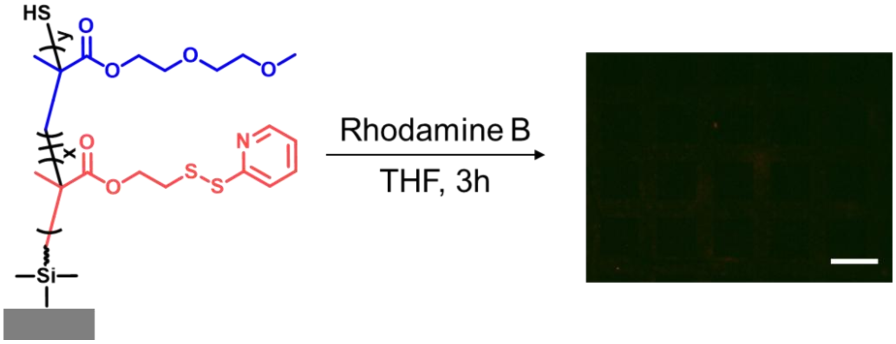

Figure S5. The possibility of the reaction of rhodamine B dye with thiol groups on the polymer chain-end.

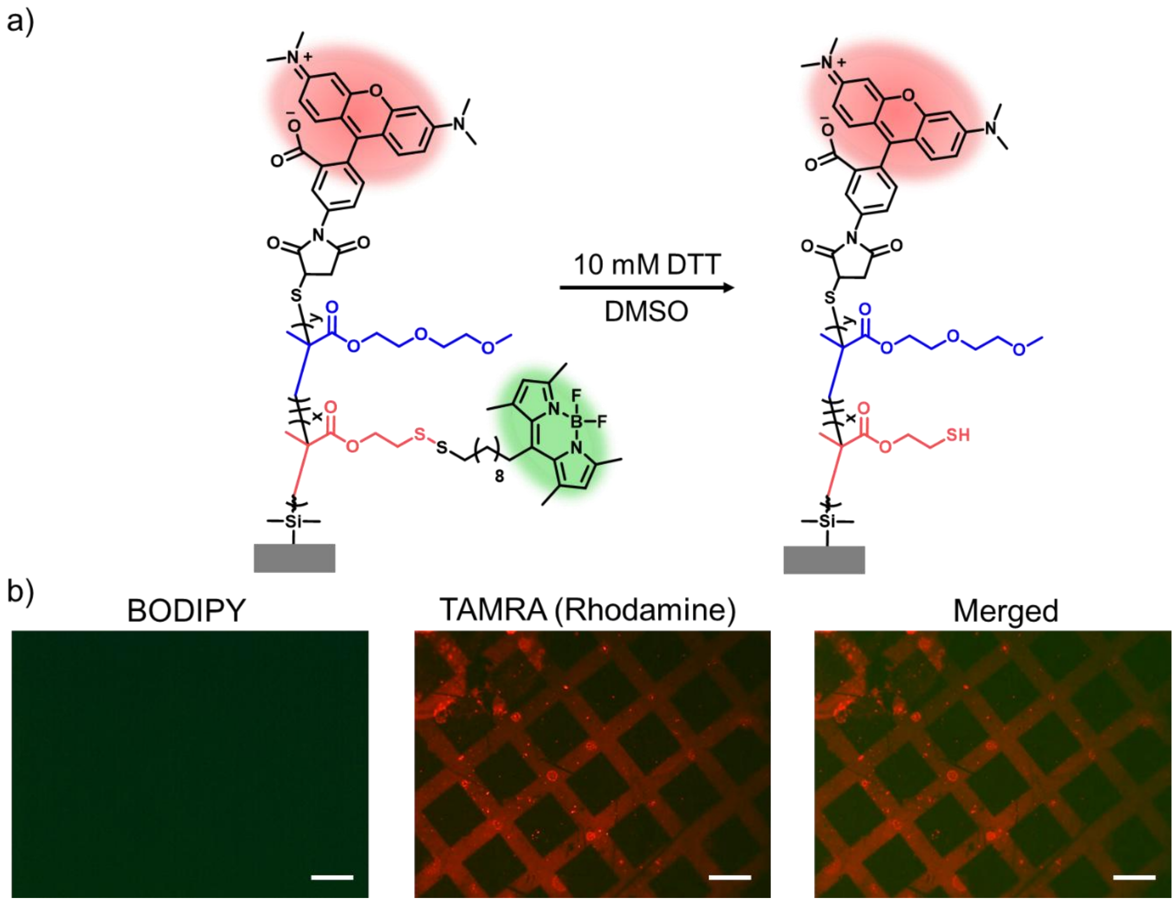

Figure S6. (a) BODIPY cleavage from P2 polymer brush with DTT. (b) The fluorescence images of rhodamine and BODIPY-conjugated P2 polymer brush after BODIPY release in the presence of DTT. The scale bar is 100 μm.

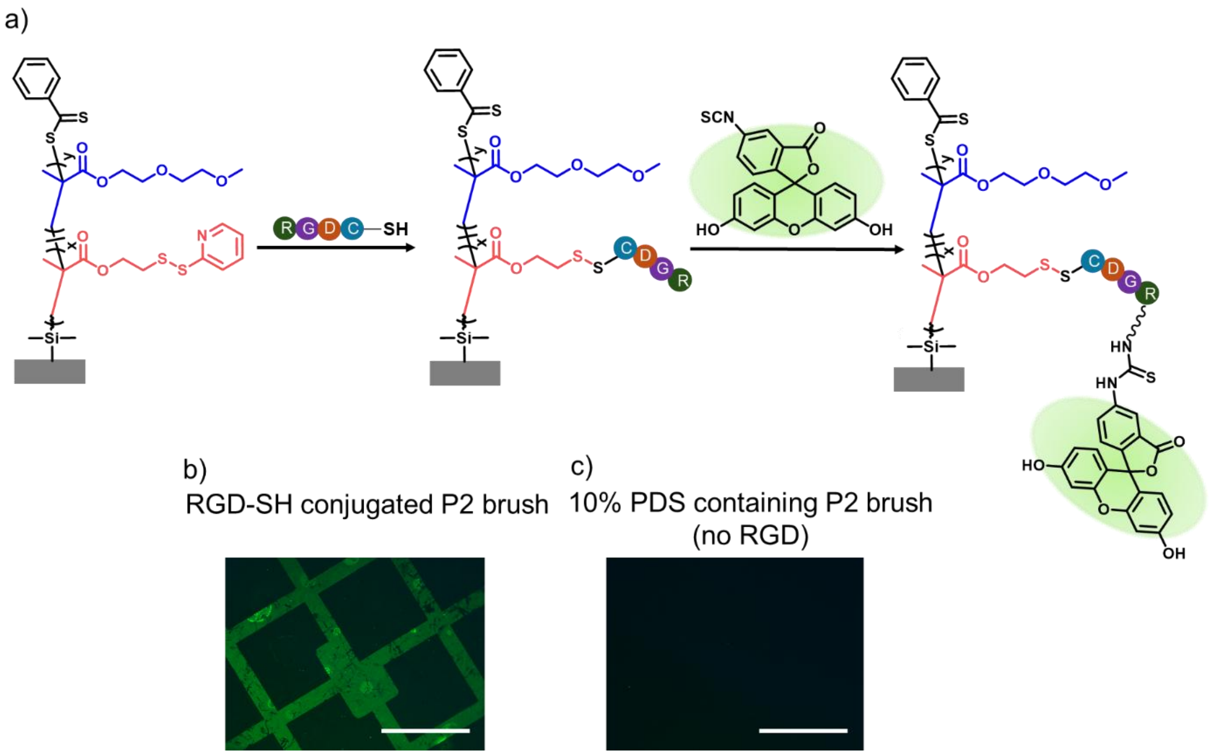

Figure S7. (a) The modification of P2 polymer brush (thickness ca. 50 nm) with thiolated RGD peptide and functionalization with FITC. (b) The fluorescence image of FITC-conjugated RGD-containing surface. (c) The fluorescence image of the control P2 surface. The scale bar is 500 μm.

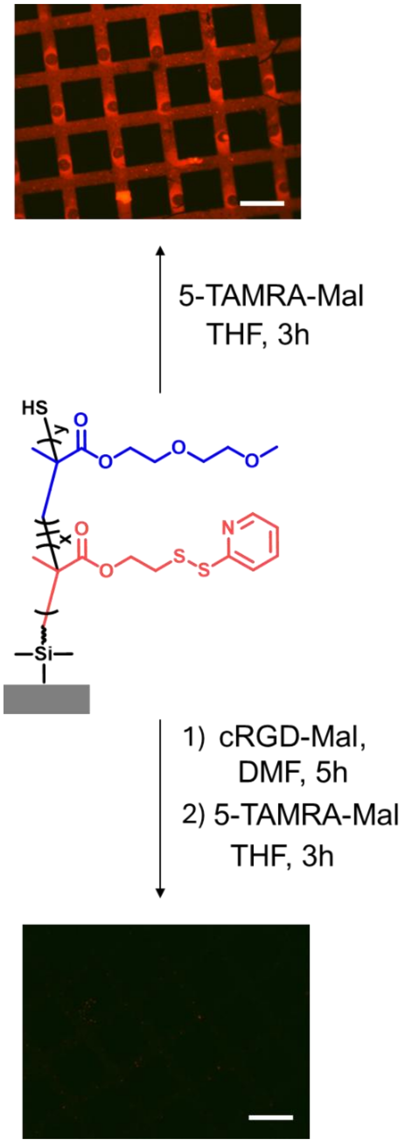

Figure S8. TAMRA-Mal reaction possibility before and after conjugation of cRGD-Mal onto P2 brush.

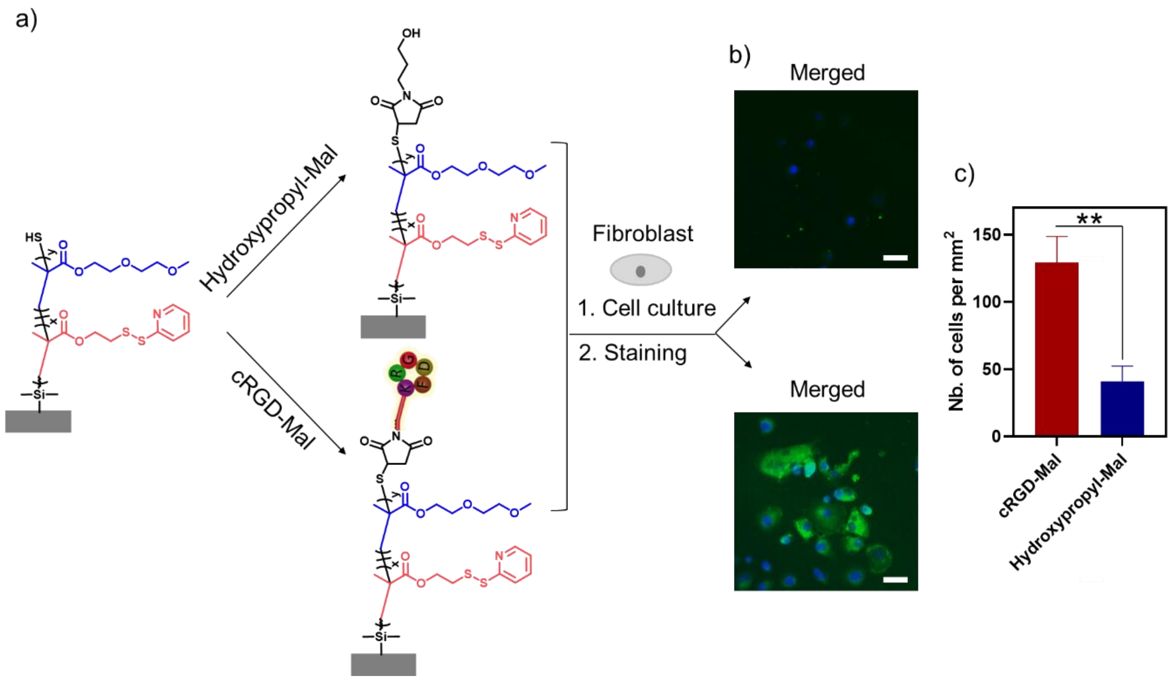

Figure S9. (a) Fabrication of cRGD-Mal and hydroxypropyl-Mal modified P2 polymer brush. (b) Fluorescence images of attached cells after staining with Alexa Fluor Phalloidin (AF488) and DAPI. (c) The number of fibroblast cells onto polymer brush surfaces per 1 mm^2. Values are average of three images taken with a 20× objective. Asterisks indicate statistically significant differences between the indicated samples **p < 0.01). The scale bar is 50 μm.

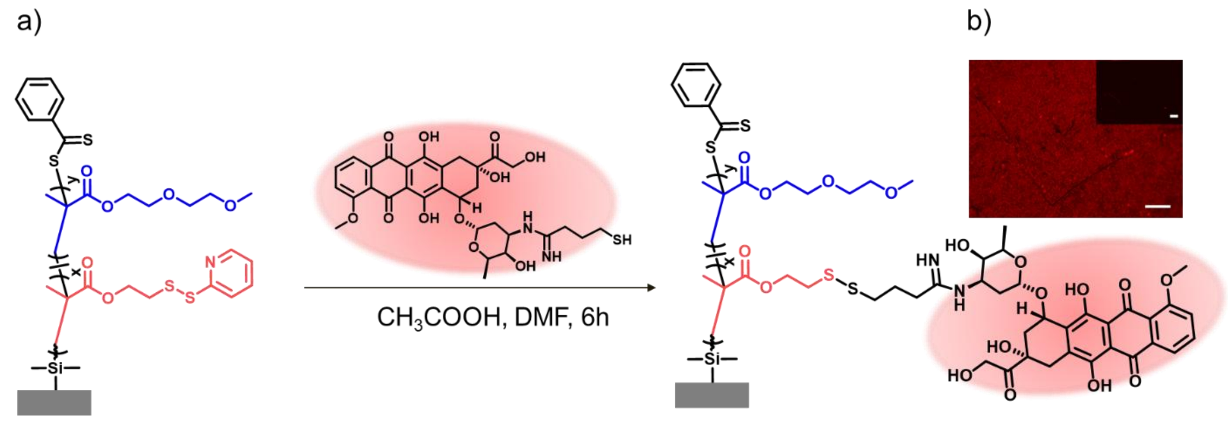

Figure S10. (a) The illustration of DOX-SH binding to polymer brush via thiol-disulfide exchange reaction, (b) the fluorescence image of P2 brush surface after modifying with DOX-SH. The scale bar is 100 μm.

Additional *In vitro* Cell Data

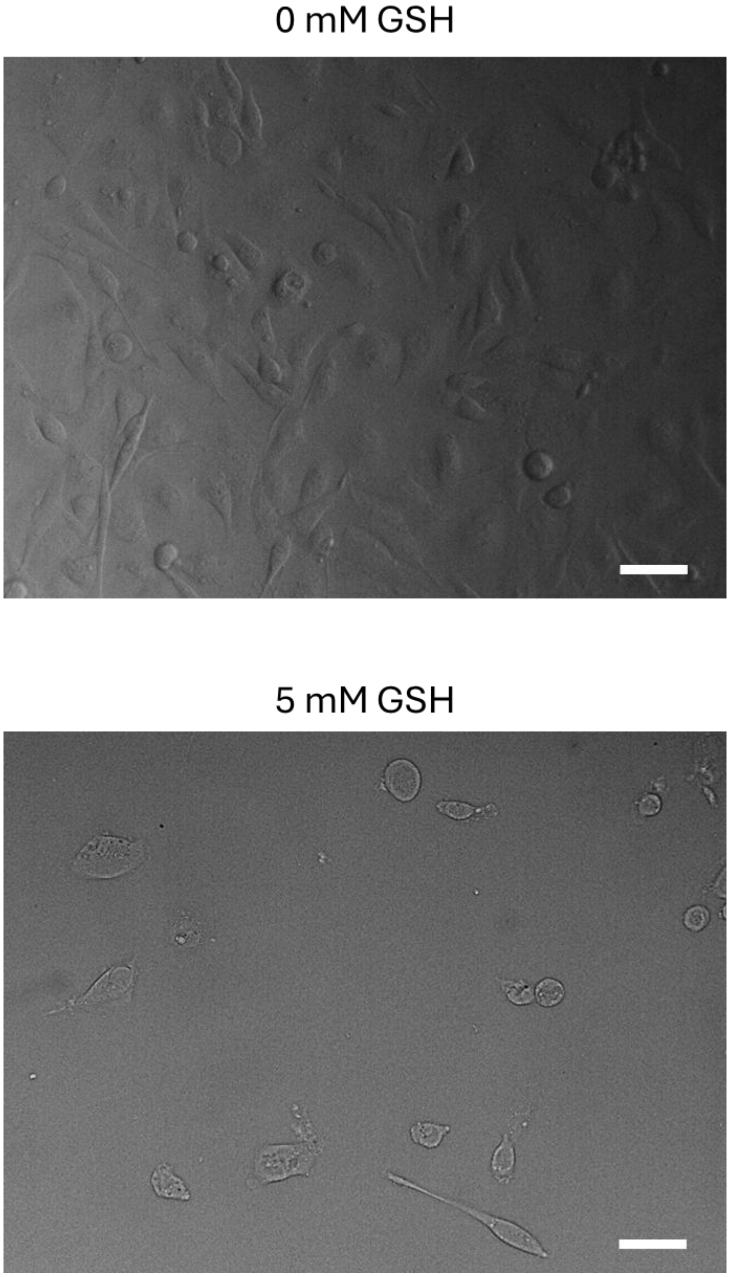

Figure S11. Bright-field images of re-seeded cells to well-plate after detachment from GSH-treated surface (5 mM GSH) and GSH-nontreated surface (0 mM GSH). The scale bar is 50 μm.

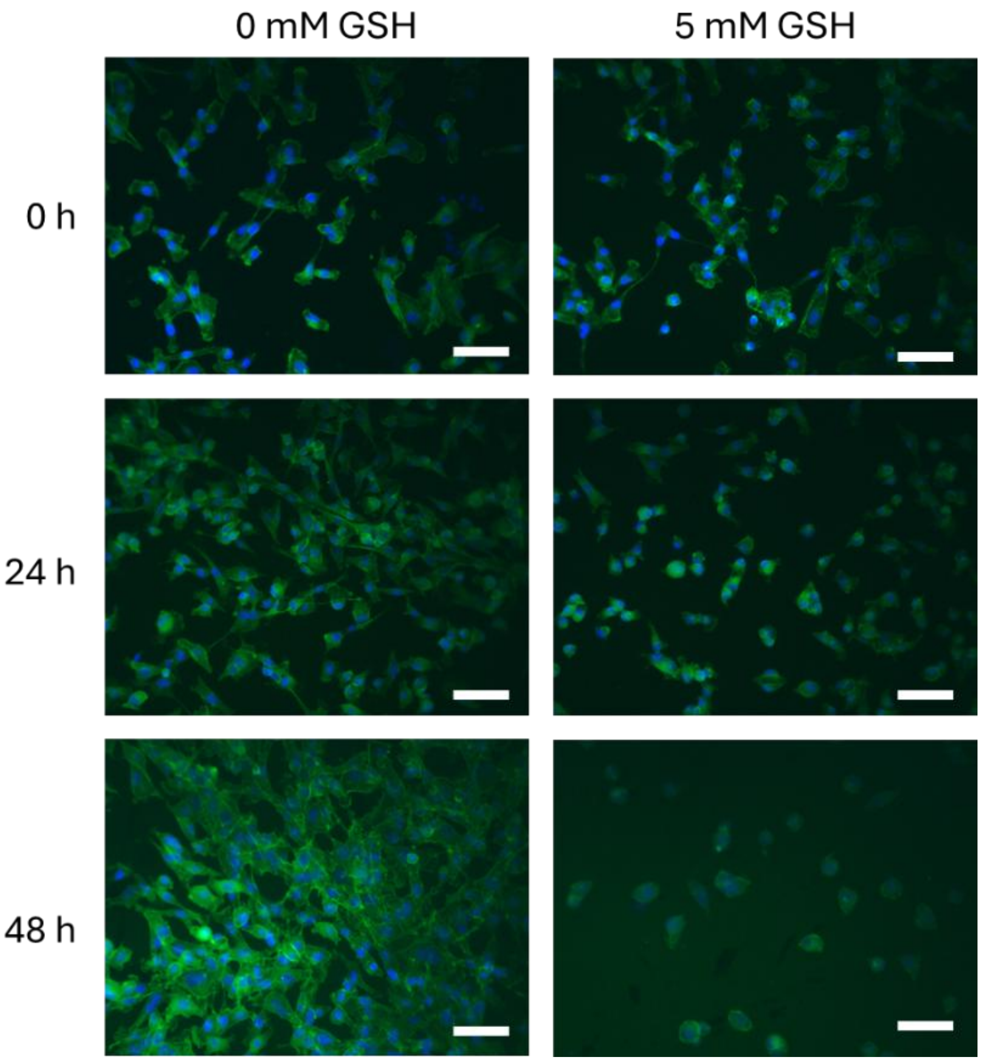

Figure S12. Proliferation of MDA-MB-231 cell on the cRGD-DOX-P2 surface with and without GSH until 48 h. Actins and nuclei of attached cells were stained by AF488 and DAPI, respectively. The scale bar is 100 μm.

References

(1) Peng, H.; Rubsam, K.; Huang, X.; Jakob, F.; Karperien, M.; Schwaneberg, U.; Pich, A. Reactive Copolymers Based on N-Vinyl Lactams with Pyridyl Disulfide Side Groups via RAFT Polymerization and Postmodification via Thiol–Disulfide Exchange Reaction. *Macromolecules* **2016**, *49*, 7141–7154.

(2) Revell, D. J.; Knight, J. R.; Blyth, D. J.; Haines, A. H.; Russell, D. A. Self-Assembled Carbohydrate Monolayers: Formation and Surface Selective Molecular Recognition. *Langmuir* **1998**, *14*, 4517–4524.

(3) Mahajan, S. S.; Iyer, S. S. ELISA and SPR Studies of Ricin Binding to β-Galactoside Analogs. *J. Carbohydr. Chem*. **2012**, *31*, 447–465.

(4) Shepherd, J. L.; Kell, A.; Chung, E.; Sinclar, C. W.; Workentin, M. S.; Bizzotto, D. Selective Reductive Desorption of a SAM-Coated Gold Electrode Revealed Using Fluorescence Microscopy. *J. Am. Chem. Soc*. **2004**, *126*, 8329–8335.

(5) Darwish, S.; Sadeghiani, N.; Fong, S.; Mozaffari, S.; Hamidi, P.; Withana, T.; Yang, S.; Tiwari, R. K.; Parang, K. Synthesis and antiproliferative activities of doxorubicin thiol conjugates and doxorubicin-SS-cyclic peptide. *European Journal of Medicinal Chemistry*, 2019, **161**, 594–606.

(6) Gunay, K. A.; Schüwer, N.; Klok, H.-A. Synthesis and post-polymerization modification of poly(pentafluorophenyl methacrylate) brushes. *Polym. Chem*. **2012**, *3*, 2186–2192.
